# Genome-wide analysis of banana MADS-box family closely related to fruit development and ripening

**DOI:** 10.1038/s41598-017-03897-1

**Published:** 2017-06-14

**Authors:** Juhua Liu, Jing Zhang, Jianbin Zhang, Hongxia Miao, Jingyi Wang, Pengzhao Gao, Wei Hu, Caihong Jia, Zhuo Wang, Biyu Xu, Zhiqiang Jin

**Affiliations:** 1 Key Laboratory of Tropical Crop Biotechnology, Ministry of Agriculture; Institute of Tropical Bioscience and Biotechnology, Chinese Academy of Tropical Agricultural Sciences, 4 Xueyuan Road, 571101 Haikou, China; 2 Key Laboratory of Genetic Improvement of Bananas, Hainan Province; Haikou Experimental Station, Chinese Academy of Tropical Agricultural Sciences, 570102 Haikou, China

## Abstract

Proteins encoded by MADS-box genes are important transcription factors involved in the regulation of flowering plant growth and development. Currently, no systematic information exists regarding the MADS-box family in the important tropical fruit banana. Ninety-six MADS-box genes were identified from the banana (Pahang) A genome. Phylogenetic analysis indicated that *Musa acuminata* MCM1-AGAMOUS- DEFICIENS-SRF (MaMADS) could be divided into MIKC^c^, MIKC*, Mα/β and Mγ groups. MIKC^c^ could be further divided into 11 subfamilies, which was further supported by conserved motif and gene structure analyses. Transcriptome analysis on the Feng Jiao (FJ) and BaXi Jiao (BX) banana cultivars revealed that *MaMADS* genes are differentially expressed in various organs, at different fruit development and ripening stages, indicating the involvement of these genes in fruit development and ripening processes. Interactive network analysis indicated that MaMADS24 and 49 not only interacted with MaMADS proteins themselves, but also interacted with hormone-response proteins, ethylene signal transduction and biosynthesis-related proteins, starch biosynthesis proteins and metabolism-related proteins. This systematic analysis identified candidate *MaMADS* genes related to fruit development and ripening for further functional characterization in plants, and also provided new insights into the transcriptional regulation of *MaMADS* genes, facilitating the future genetic manipulation of MADS-mediated fruit development and ripening.

## Introduction

MADS-box proteins are important transcription factors involved in the regulation of flowering plant growth and development ranging from the roots to the flowers and fruits. Plant MADS-box genes can be classified into two categories, type I and type II, based on their evolutionary origin. Type I MADS transcription factors are further divided into Mα, Mβ and Mγ groups based on the M domain of the encoded protein, although their function has yet not been fully characterized. Type II MADS transcription factors can be divided into the MEF2-like group existing in animals and yeast, and the MIKC group, which is present only in plants^1–3^. To date, MIKC-type MADS-box genes have been found in several plants, including 75 (37 type I and 38 type II) in rice^4^, 107(68 type I and 39 type II) in Arabidopsis^2^, 105 (41 type I and 64 type II) in poplar^5^, 58 (20 type I and 38 type II) in grape^6^, 43 (13 type I and 30 type II) in cucumber^7^ and 106 (34 type I and 72 type II) MADS-box genes in soybean^8^, respectively. Based on differences in their domain structure, MIKC-type MADS-box genes have been classified into MIKC^C^ and MIKC* types^9^. The latter are characterized by an altered protein domain structure that is possibly linked to a duplication of exons encoding the K domain subregion^10^. Based on their phylogenetic relationships, MIKC^C^-type MADS-box genes can be divided into 13 distinctive subfamilies known as AG, AGL6, AGL12, AGL15, AGL17, BS/GGM13, DEF/GLO, FLC, SEP/AGL2, AP1/FUL, STMADS11/SVP, TM3/SOC1 and TM8 based on their phylogeny. The full names of these subfamilies were listed in Supplementary Table S7. Most MIKC^C^-type gene subfamilies appear to have originated in ancestral seed plants and were named after their founding members^11, 12^.

MADS-box genes play important roles in nearly every aspect of plant growth and development^13–15^. Current evidence suggests that MADS-box transcription factors (TFs) are the most powerful TFs in regulating fruit development and ripening^16^. Most investigations on the function of MADS-box TFs on fruit development and ripening have focused on the model plant tomato (*Solanum lycopersicum*), while little research has focused on other plants, particularly the important tropical fruit, banana. Moreover, the reports concerning the role of MADS-box in the control of banana fruit development and ripening have been scattered^17–21^ and no systematic information is available regarding the MADS-box family in banana.

With respect to trade volume, banana (*Musa acuminata*) is the world’s most important fresh fruit commodity contributing to about 16% of the world’s total global fruit production^22^. In comparison to other fresh fruits, the development and ripening of banana fruit has its own characteristics. Firstly, flowering is inseparable to fruit development. Secondly, seedless fruits develop parthenocarpically from the female flowers with numerous aborted ovules. Thirdly, the distinction between fruit development and ripening is well-defined. The fruit is filled with starch during pre-harvest fruit development, which is then partially converted to sugars during the post-harvest ripening process^23^. These characteristics attracted our attention to the role MADSes work during banana fruit development and ripening. The completion of the genome sequencing of the banana (DH-Pahang) A made a genome-wide analysis of MADS-box genes possible^24^. Due to the significance of MADS-box transcription factors in various aspects of biological processes, especially their crucial roles in fruit development and ripening, we chose the MADS-box genes family for a systematic analysis in banana.

## Results and Discussion

### Identification and evolutionary analysis of *MaMADSes* in banana

**Figure 1:**
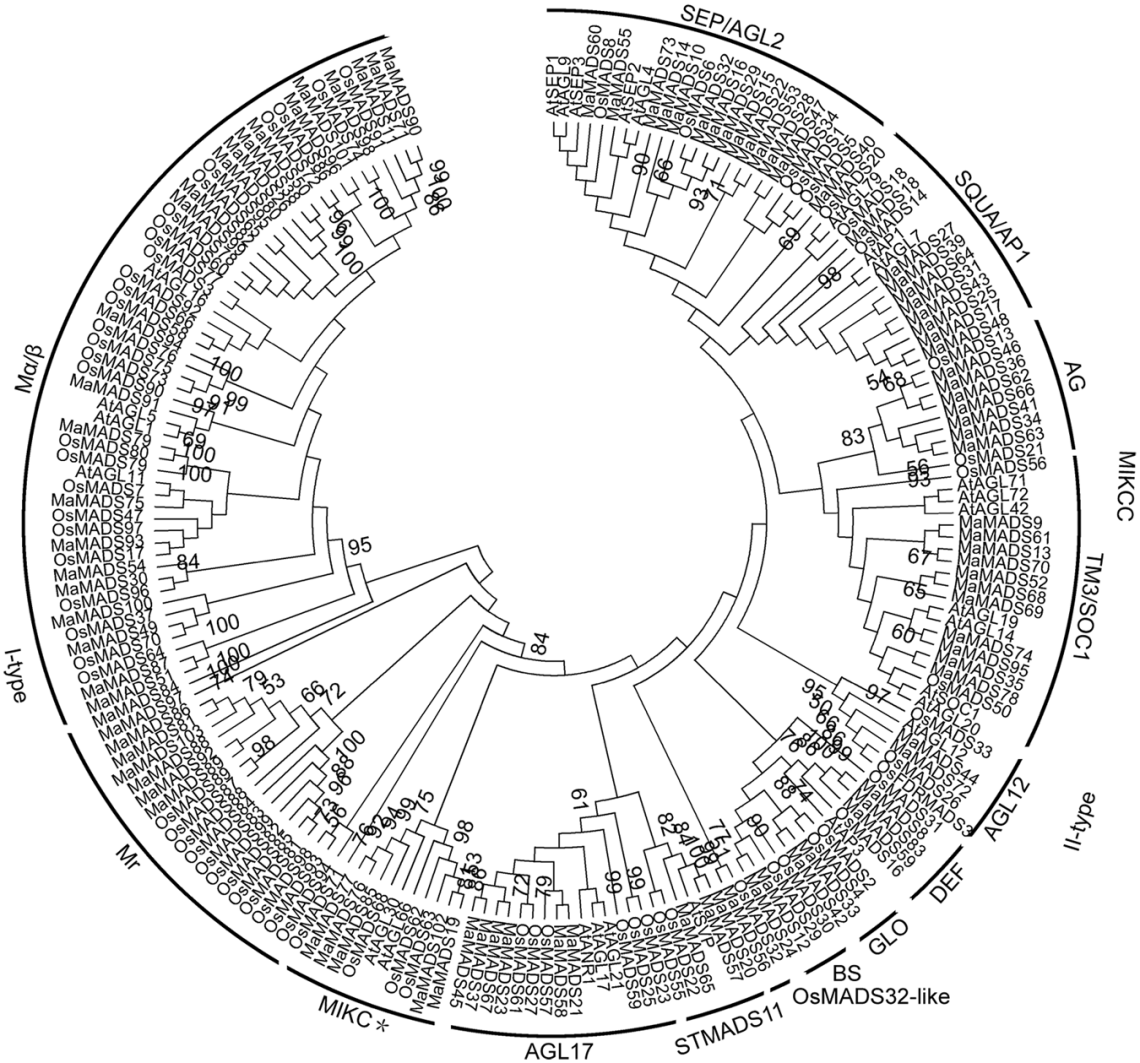
Phylogenetic analysis of the MADSes from *Arabidopsis*, rice and banana. The Neighbor-joining (NJ) tree was drawn using MEGA 5.0 with 1000 bootstrap.

To extensively identify banana MADS-box genes, we used BLAST and Hidden Markov Model searches to explore the banana genome database using MADS-box sequences from Arabidopsis and rice as queries. Following this, 96 putative MADS-box members were characterized from banana, and further conserved domain detection confirmed that all the identified *Musa acuminata* MCM1-AGAMOUS-DEFICIENS-SRF (MaMADSes) harbored the conserved MADS domain that is the basic characteristic of the MADS-box family. The 96 predicted banana MADS-box proteins varied from 63 (MaMADS95) to 527 (MaMADS80) amino acid residues and the relative molecular mass ranged from 7.2 (MaMADS95) to 56.6 (MaMADS80) kDa, with isoelectric points in the range of 5.42–10.82 (Supplementary Table S1). To explore the evolutionary relationships between MaMADSes from banana and other known MADSes from Arabidopsis and rice, an unrooted Neighbor-Joining tree was created (Fig. 1; Supplementary Table S1). The results showed that the 96 *MaMADSes* were localized on 11 chromosomes based on the genome database (http://banana-genome.cirad.fr/) (released in 2016). Fourteen genes (the maximum: 14.6%) were localized on chromosome 3, followed by 12 (12.5%) on chromosome 11 and 11 (11.5%) on chromosomes 5, whereas chromosome 1 had only four MADS-box genes. These 96 *MaMADSes* could be assigned to two groups, namely type I (31) and type II (65). Type I could then be divided into Mγ (8) and Mα/β (23). Type II could be divided into MIKC* (5) and MIKC^c^ (60). MIKC^c^ could be further divided into 11 subfamilies, together with their orthologous MADSes from Arabidopsis and rice. Subfamily OsMADS32-like contained only one MaMADS protein, whereas subfamily SEP/AGL2 had the maximum MaMADS members (14), followed by 11, nine, seven, six, three, three, two, two and two members for subfamilies TM3/SOC1, AP1/FUL, AG, AGL17, STMADS11/SVP, DEF, AGL12, GLO and BS, respectively. Representatives of banana, rice and Arabidopsis could be identified in all the clades except for the TM8, FLC, AGL6 and AGL15, which are exclusive to banana (Fig. 1). This suggests that these subfamilies exhibit little conservation and that their functions are performed by other genes^8^. Moreover, unlike rice and Arabidopsis, the Mα/β clade could not be clearly divided into Mα and Mβ, suggesting that these genes were very closely linked during their long evolutionary history. Generally, MaMADSes from banana were found to be more closely associated with the MADSes from rice than that from Arabidopsis. This is probably because that banana and rice are both monocotyledons. Evolutionary analysis also identified some closely related orthologous MADSes between banana and rice, indicating that an ancestral set of *MADSes* genes existed prior to the divergence of banana and rice.

**Figure 2:**
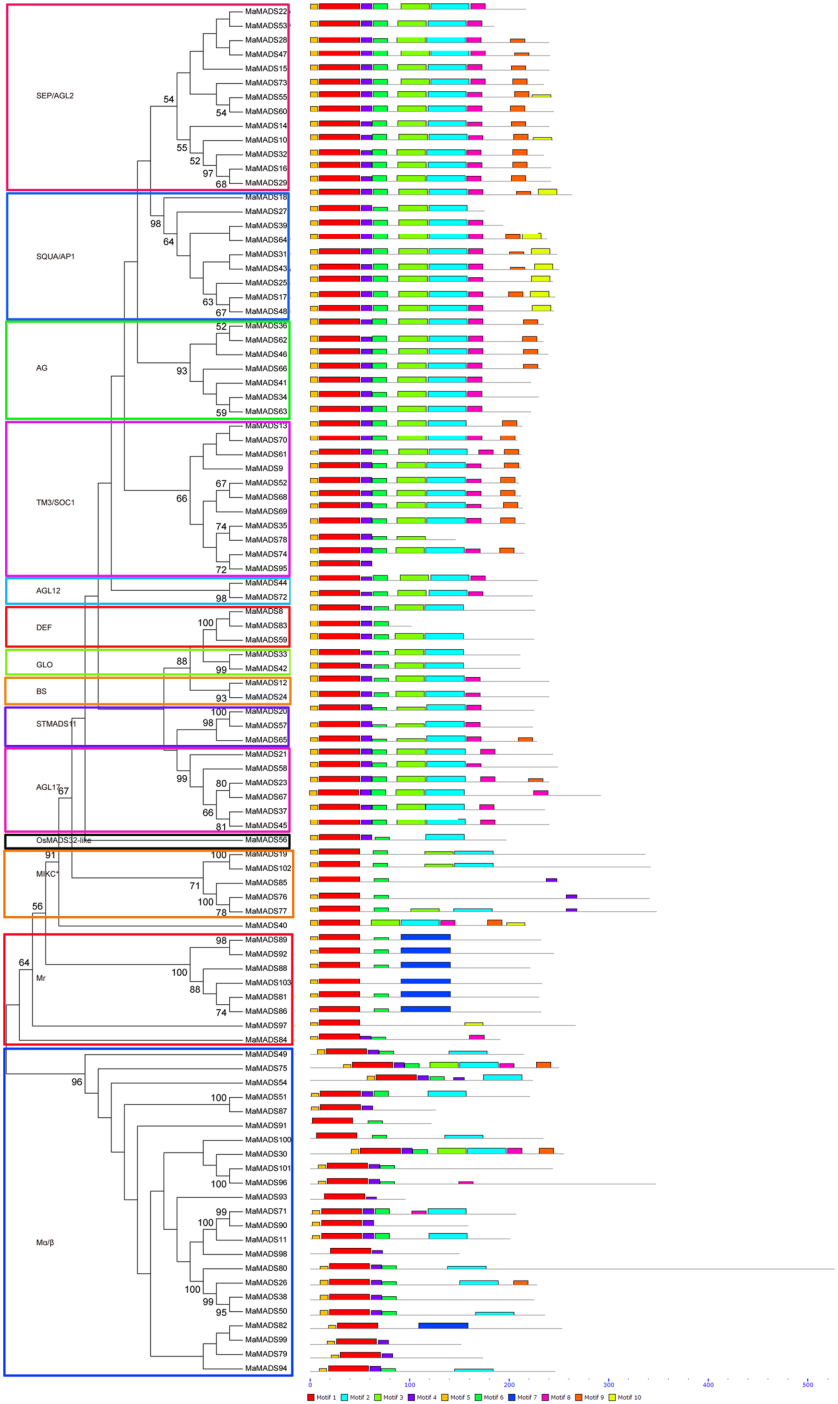
Conserved motif analyses of MaMADSes. All motifs were identified by MEME database with the complete amino acid sequences of MaMADSes. The classification of MaMADSes were shown based on the phylogenetic relationship.

**Figure 3:**
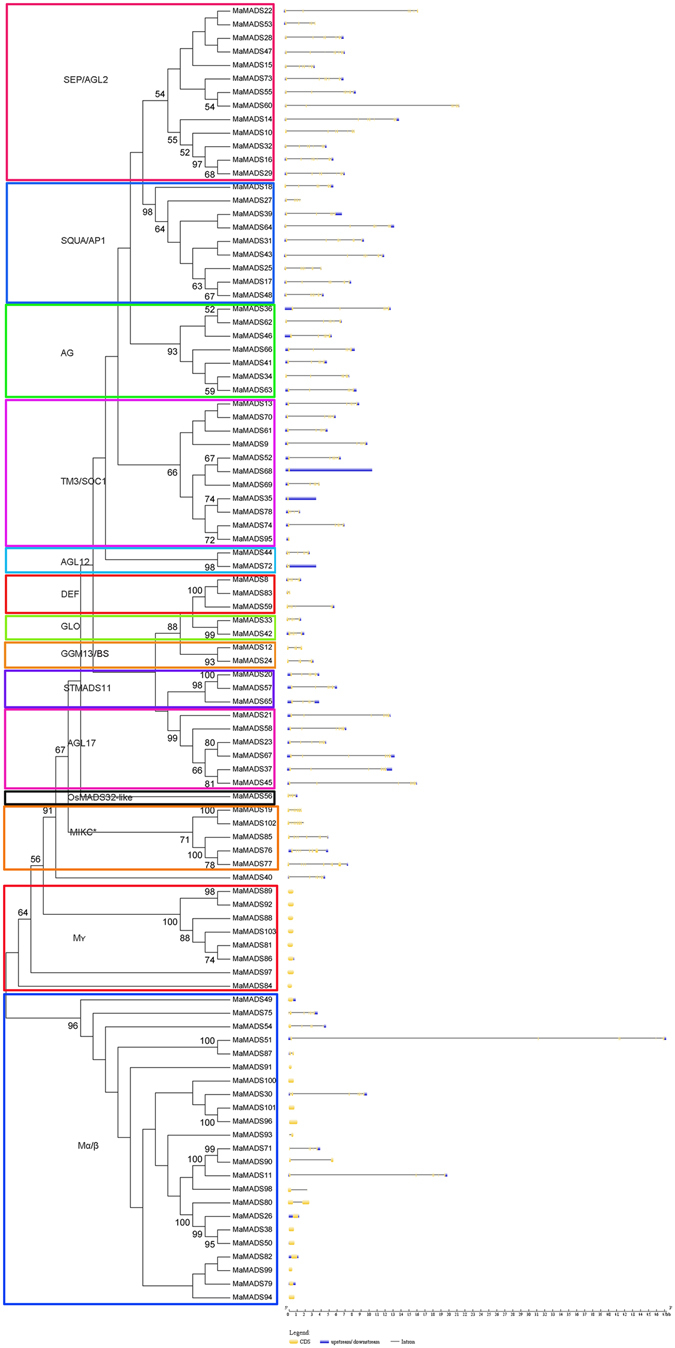
Gene structure analyses of *MaMADSes*. Exon-intron structure analyses were performed by GSDS database. The blue boxes indicate upstream/downstream, the yellow boxes indicate exons, and the black lines indicate introns.

To obtain insight into the divergence and function of the MaMADSes proteins, a total of 10 conserved motifs were captured by MEME software and annotated with the InterPro database (Fig. 2). All the MaMADSes contained the basic MADS domain of motif 1, and most of the MaMADSes, with the exception of the Mγ and partial Mα/β group, also contained the basic K-box domain of motif 2 and motif 3. Most of the MaMADSes (excepting mainly for the MIKC* and Mγ group) and almost all the MaMADSes (except for MaMADS91, 93, 98, 100) contained the motif 4 and 5, respectively, which was annotated as MADS box 2. Seven MaMADSes including MaMADS81, 86, 88, 89, 92 and 103 belonged to Mγ and 82 belonged to Mα/β and contained motif 7. The motif analyses showed that most conserved motifs existed in the same group. Conserved MADS-box motifs have also been observed in other plants such as grapevine^25^ and rice^4^. Furthermore, simpler functional roles are also associated with lower domain complexities. For example, few functions have been reported regarding MIKC*, Mγ and Mα/β on plant growth and development, and their structures are relatively simple with containing three to five motifs. However, the majority of the MIKC^c^ group including 11 subfamilies with numerous functions in plant growth and development contain complicated structures with seven to nine motifs. These results suggest that most of the MaMADSes that clustered in the same group share similar amino acid sequences, which further corroborates the banana MaMADSes phylogenetic analyses. Exon-intron organizations of the 96 *MaMADSes* genes were also examined in an attempt better understand their structural evolution. As shown in Fig. 3, only 20 out of a total of 96 *MaMADSes* have one exon, and most of the intronless genes were clustered in the type I group. The other *MaMADSes* contained exons with numbers varying from two to eight, and most of the genes with seven to eight exons were grouped in the MIKC^c^ group. This suggested that similar exon-intron organizations of *MaMADSes* exist in the same group and that the gene structure might be meaningful for gene evolution and function. According to a previous report on rice, the rate of intron loss is faster than the rate of intron gain following segmental duplication^26^. Thus, it can be concluded that most of the II-type MADS-box genes might contain the original genes, and most of the I-type group appear to be predominantly duplicated via segmental duplications with subsequent intron loss. This *MaMADSes* gene structure feature has also been observed in other plants, such as rice, cucumber and grape^4, 7, 25^. However, several type I group genes such as *MaMADS75*, *54*, *51*, *30*, *71*, *11* and *80* also have intro structure, indicating these genes might have inequable functions.

### Expression profiles of *MaMADSes* genes in different organs of two banana varieties

**Figure 4:**
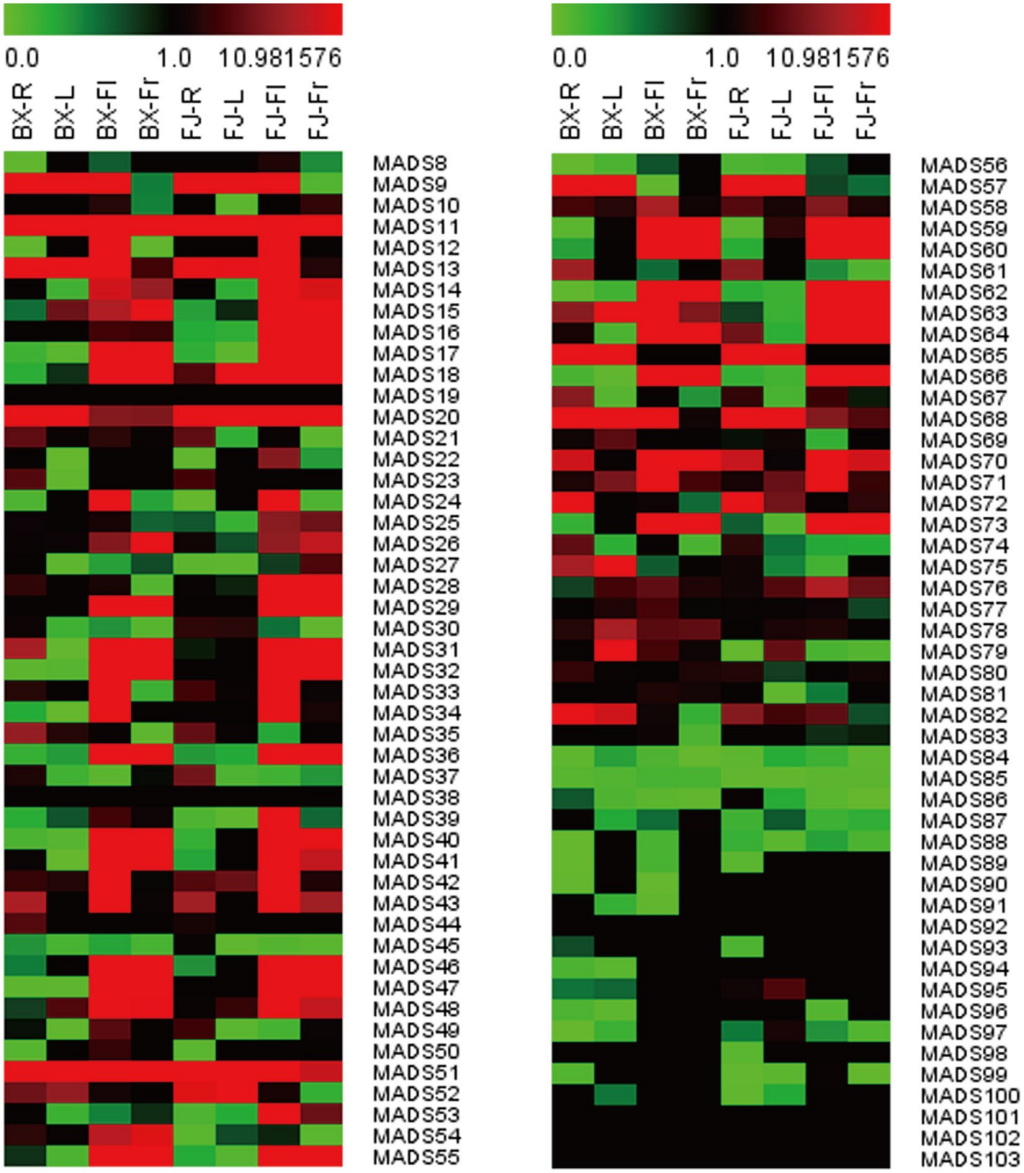
Expression patterns of *MaMADSes* in the roots (R), leaves (L), flowers (Fl) and fruits (Fr) of BX and FJ. The heat map with clustering was created based on the FPKM value of *MaMADSes*. Differences in gene expression changes are shown in color as the scale.

To investigate the organ expression patterns of MADS genes in banana, the roots, leaves, flowers and fruits of the BaXi Jiao (BX) and Fen Jiao (FJ) varieties were sampled for RNA-seq assays. Among the 96 *MaMADSes* genes, 90 genes (except for *MaMADS19*, *38*, *92*, *101*, *102*, *103*) showed expression in at least one tested organ of the two varieties (Fig. 4; Supplementary Table S2). For BX, 77 (85.5%), 67 (74.4%), 77(85.5%) and 64 (71.1%) *MaMADSes* were expressed in the roots, leaves, flowers and fruits, respectively, among which 10 (13.0%), 11 (16.4%), 32(41.6%) and 24 (37.5%) genes showed high expression levels (value >10), and 0 (0%), 0 (0%), 10 (31.2%) and eight (33.3%) genes displayed extremely high expression levels (value >100), respectively. In particular, the expression values of *MaMADS36* (AG subfamily), *55* (SEP/AGL2 subfamily) and *73* (SEP/AGL2 subfamily) in the flowers and fruits were greater than 400. The most highly expressed gene in the flowers was *MaMADS46* (AG subfamily), which reached a value of 1,091, while the most highly expressed in the fruits was *MaMADS55* (SEP/AGL2 subfamily), which reached a value of 896. Additionally, only two (2.2%) genes (*MaMADS11* and *51*) belonging to the Mα/β subfamily had high transcriptional expression levels (value >10) in all of the four tested organs. For FJ, 77 (85.5%), 64 (71.1%), 79 (87.8%) and 71 (78.9%) *MaMADSes* were expressed in the roots, leaves, flowers and fruits, respectively, among which 10 (13.0%), 10 (15.6%), 38 (48.1%) and 23 (32.4%) genes showed high expression levels (value >10), and 0 (0%), 3 (0%), 12 (31.6%) and six (26.1%) genes displayed extremely high expression levels (value >100), respectively. The expression values of *MaMADS55* (SEP/AGL2 subfamily) in the flowers and fruits were in excess of 400. The most highly expressed gene in the flowers was *MaMADS46* (AG subfamily), which reached 846, while and the most highly expressed gene in the fruits was *MaMADS60* (SEP/AGL2 subfamily), which reached 824. Additionally, only two (2.2%) genes (*MaMADS11* and *51*) belonging to the Mα/β subfamily had high transcriptional expression levels (value >9) in all of the four tested organs. These results indicated that the MADS-box genes were differentially expressed in the different banana organs. The high expression levels and gene number in flowers and fruits suggested that *MaMADSes* play more important roles in the flowers and fruits than in the other organs, which is consistent with a previous report that suggested that floral organ identity is governed by MADS-box transcription factors in accordance with the extended ABC model^16^. For example, the knock-down of *TOMATO AGAMOUS 1* (*TAG1*) by RNA interference (RNAi) results in stamen defects and loss of determinacy, leading to nested flowers-in-flowers^27^ or a fruit-in-fruit phenotype^28^. The knock-down of the tomato SHP orthologue *TOMATO AGAMOUS-LIKE 1* (*TAGL1*) appears to affect carpel identity by leading to loss of style trichomes and a thinner fruit pericarp^29^.

Almost all of the *MaMADSes* showed similar expression patterns to the organ expression characteristics between BX and FJ, with more *MaMADSes* genes showing high expression levels in the flowers and fruits than in the roots and leaves. The 21 genes that were highly expressed in the flowers and fruits included 12 SEP/AGL2 genes (*MaMADS10*, *14*, *15*, *16*, *28*, *29*, *32*, *40*, *47*, *55*, *60* and *73*); three AP1/FUL genes (*MaMADS17*, *18* and *64*); and six AG genes (*MaMADS36*, *41*, *46*, *62*, *63* and *66*). This is consistent with the previously reported that found that the TFs of the MADS-box subfamilies SEP, AG and FUL control fleshy fruit development and ripening^16^. Two DEF genes (*MaMADS59* and *83*) and one Mα/β gene (*MaMADS26*) were highly expressed in the flowers and fruits in both BX and FJ, indicating that banana fruit development and ripening is a complicated process that requires the participation of other subfamilies. Five genes (two BS genes, *MaMADS12* and *24*; one GLO gene, *MaMADS33*; one AG gene, *MaMADS34* and one AP1/FUL gene, *MaMADS39*) were specially expressed in the flowers of both BX and FJ. Three genes (one STMADS11/SVP gene, *MaMADS23*; one AGL12 gene, *MaMADS44* and one TM3/SOC1 gene, *MaMADS61*) were specially expressed in the roots of both BX and FJ. Seven genes (four TM3/SOC1 genes, *MaMADS9*, *35*, *52* and *69*; one OsMADS32-like gene, *MaMADS56*; one GLO gene, *MaMADS42* and one Mα/β gene, *MaMADS82*) were highly expressed in the roots, leaves and flowers of both BX and FJ. Five genes (two Mα/β genes, *MaMADS30* and *75*; two STMADS11/SVP genes, *MaMADS65* and *57*; one TM3/SOC1 gene, *MaMADS95*) were highly expressed in the roots and leaves of both BX and FJ. *MaMADS43* (AP1/FUL) was highly expressed in the roots, flowers and fruits of both BX and FJ. *MaMADS48* (AP1/FUL) and 53 (SEP/AGL2) were highly expressed in the leaves, flowers and fruits of both BX and FJ. *MaMADS67* (AGL17) was highly expressed in the roots and flowers of both BX and FJ, and *MaMADS79* (Mα/β) was highly expressed in the leaves of both BX and FJ. These results suggest that *MaMADSes* genes play different roles in the different organs of the two banana varieties. Additionally, 19 genes (six Mα/β genes, *MaMADS11*, *51*, *54*, *71*, *80* and *84*; one Mγ gene, *MaMADS86*; three MIKC* genes, *MaMADS76*, *77* and *85*; five TM3/SOC1 genes, *MaMADS13*, *68*, *70*, *74* and *78*; two AGL17 genes, *MaMADS45* and *58*; one STMADS11/SVP gene, *MaMADS20*, and one AGL12 gene, *MaMADS72*) were constitutively expressed in both BX and FJ, indicating their important roles in organ development. Interestingly, we found three Mα/β genes (*MaMADS11*, *26* and *51*) and two DEF genes (*MaMADS59* and *83*) that were highly expressed in the fruits of both BX and FJ, suggesting that these genes might play important roles in the banana fruit ripening process. Together, these organ expression profiles of the *MaMADSes* genes in different varieties may provide insight for future studies on organ development and function.

### Expression profiles of *MaMADSes* genes in different stages of fruit development and ripening of two banana varieties

**Figure 5:**
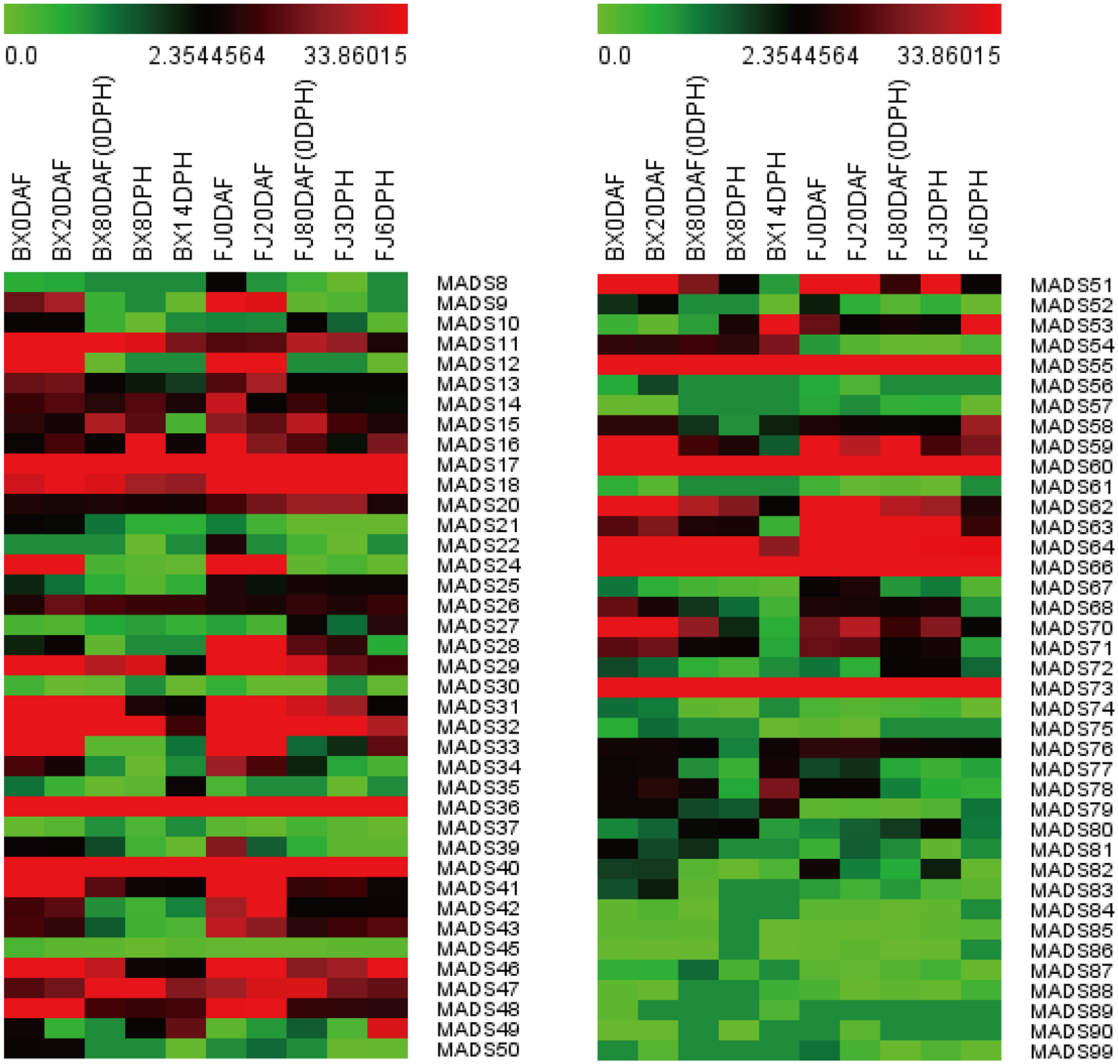
Expression patterns of *MaMADSes* in different stages of fruit development and ripening in BX and FJ varieties. The heat map with clustering was created based on the FPKM value of *MaMADSes*. Differences in gene expression changes are shown in color as the scale.

To understand roles of the *MaMADSes* genes in the development and ripening of banana fruit, their expression patterns were detected in fruits sampled at 0, 20 and 80 days after flowering (DAF). Additionally, the expression patterns in the fruits at 0, 8 and 14 days post-harvest (DPH) were detected in BX, and at 0, 3 and 6 DPH in FJ (Fig. 5; Supplementary Table S3). For BX, among the 96 *MaMADSes*, 78 genes (excluding *MaMADS19*, *23*, *38*, *44*, *65*, *69*, *91*, *92*, *93*, *94*, *95*, *96*, *97*, *98*, *100*, *101*, *102* and *103*) were expressed at different stages of fruit development and ripening. Seventy-six (97.4%), 76 (97.4%), 64 (82.1%), 59 (75.6%), 60 (76.9%) *MaMADSes* were expressed at 0 DAF, 20 DAF, 80 DAF (0 DPH), 8 DPH and 14 DPH, among which 32 (41.6%), 32 (40.0%), 23 (35.9%), 17 (28.8%) and 17 (27.0%) genes, respectively, showed high expression levels (value >10) at each stage. Of these, nine (28.1%), nine (28.1%), nine (39.1%), six (35.3%) and five (29.4%) genes were extremely highly expressed (value >100). Moreover, the most highly expressed genes at 0 DAF, 20 DAF, 80 DAF (0 DPH), 8 DPH and 14 DPH were *MaMADS46* (AG), *46* (AG), *55* (SEP/AGL2), *73* (SEP/AGL2) and *73* (SEP/AGL2), respectively, which reached values of 1,091, 1,142, 896, 855 and 1,038. For FJ, 76 (97.4%), 74 (94.9%), 70 (89.7%), 72 (92.3%), 66 (84.6%) *MaMADSes* were expressed at 0 DAF, 20 DAF, 80 DAF (0 DPH), 3 DPH and 6 DPH, among which 38 (50.0%), 35 (47.3%), 23 (32.9%), 24 (33.3%) and 20 (30.3%) genes, respectively, were highly expressed (value >10) at each stage. Of these, 12 (31.6%), 11 (31.4%), seven (30.4%), six (25.0%) and five (25.0%) were extremely highly expressed (value >100). Additionally, the most highly expressed genes at 0 DAF, 20 DAF, 80 DAF (0 DPH), 3 DPH and 6 DPH were *MaMADS46* (AG), *55* (SEP/AGL2), *55*(SEP/AGL2), *60* (SEP/AGL2) and *73* (SEP/AGL2), respectively, which reached to 846, 672, 864, 653 and 2,643. A comparison of the expression profiles of the *MaMADSes* at distinct stages of fruit development and ripening in BX and FJ indicated that 50 genes were expressed at all stages tested in BX, whereas 62 genes were expressed at all stages tested in FJ, which is 1.24-fold higher than that in BX. It appears that the mechanism of fruit development and ripening in FJ is more complex than BX, and that the MADS family also possessed functions more important to development and ripening in FJ than BX (Fig. 5, Supplementary Table S3). The majority of edible cultivated bananas are triploid with a genome constitution of AAA, AAB and ABB, originating from intraspecific or interspecific hybridization between wild diploid *Musa acuminata* (A-genome) and *Musa balbisiana* (B-genome) species^30, 31^. Owing to both natural and human selection, the BX (AAA) is associated with high fruit quality, which is determined by pre-harvest development and post-harvest ripening processes^32^. However, FJ (AAB) displays interesting agronomic characteristics such as rapid fruit maturation and a high rate of starch conversion (data not shown), resulting in better flavor and taste. Additionally, it only takes six days for the FJ fruits to ripen post-harvest, which is eight days earlier than that of BX (see Materials). Taken together, this suggests that the high ratio of *MaMADSes* genes with high expression levels in different fruit developmental and ripening stages in FJ might contribute greatly to fruit ripening and quality formation. A total of 16 genes (two Mα/β genes, *MaMADS11* and *26*; one MIKC* gene, *MaMADS76*; one STMADS11/SVP gene, *MaMADS20*; two AG genes, *MaMADS36* and *66*; three AP1/FUL genes, *MaMADS17*, *18* and *64*; and seven SEP/AGL2 genes, *MaMADS29*, *32*, *40*, *47*, *55*, *60* and *73*) exhibited high transcript accumulation (value >10, with the exception of *MaMADS20* and *26*) at all stages of fruit development and ripening in both BX and FJ. This suggests that these genes might play extensive and vital roles in banana developmental and ripening processes. A total of 32 genes (seven Mα/β genes, *MaMADS30*, *50*, *51*, *54*, *71*, *75* and *82*; one MIKC* gene, *MaMADS77*; one OsMADS32-like gene, *MaMADS56*; two DEF genes, *MaMADS8* and *59*; two GLO genes, *MaMADS33* and *42*; four TM3/SOC1 genes, *MaMADS9*, *13*, *52* and *70*; one AGL17 gene, *MaMADS58*; two BS genes, *MaMADS12* and *24*; two SEP/AGL2 genes, *MaMADS10* and *28*; six AG genes, *MaMADS34*, *36*, *41*, *46*, *62* and *63*; and four AP1/FUL genes, *MaMADS31*, *39*, *43* and *48*) were mainly expressed at fruit developmental stages in both BX and FJ, among which 20 (62.5%) non-SEP/AGL2, AG and AP1/FUL genes were mainly expressed at fruit development stages, suggesting these genes might be involved in the banana fruit development process. Two genes *MaMADS49* (Mα/β) and *53* (SEP/AGL2), were mainly expressed during fruit ripening stages, suggesting these genes might play important roles in fruit ripening in banana. Generally, the *MaMADSes* expression profiles were similar in BX and FJ, with more highly expressed genes (value >10) at 0 and 20 DAF compared with the other stages, indicating that the *MaMADSes* are important in the early fruit developmental stages of 0 and 20 DAF in both BX and FJ. This phenomenon is likely attributed to the fact that banana flowering is inseparable to fruit development, and the original functions of MADS-box genes in flowering also act on fruit development^23^. In addition, *MaMADS25* (AP1/FUL), *27* (AP1/FUL), *28* (SEP/AGL2) and *63* (AG) were specially and highly expressed during the fruit development and ripening stages in FJ, while *MaMADS54* (Mα/β) were specially and highly expressed during the fruit development and ripening stages in BX, suggesting gene plasticity^16^.

Members of the different MADS-box subfamilies often have related or even conserved functions in different flowering plant species. In this study, most of the genes that were highly expressed during the processes of fruit development and ripening belonged to the SEP/AGL2 (10), AG (8) and AP1/FUL (7) subfamilies. This is consistent with previous findings that the specification of the flower types involved in the differential development of stamens and carpels and their effect on fruit and seed development are exerted by genes of the AG, AP1/FUL and SEP/AGL2 clades in different angiosperm species^12, 16^. Similarly, members of the DEF and GLO subfamilies control stamen and petal identity^33^. Other MIKC^c^ genes were later identified as being involved in different regulatory networks controlling flowering time and flower initiation: *SOC1* and *STMADS11*/*SVP* act predominantly in floral transition via the integration of signals from different flowering time regulatory pathways. Together with other subfamilies such as *AGL12* and *AGL17* ^25^, these genes function as either positive (*SOC1*) or negative regulators (*SVP*) of flower meristem identity genes. In the present study, four TM3/SOC1, two BS, two DEF, two GLO, one STMADS11/SVP, and one AGL17 *MaMADSes* were highly expressed during fruit developmental and ripening processes, indicating their novel roles rather than functioned on flower. Unfortunately, reports concerning the function of MIKC*-type and I-type MADS-box genes are limited. Early reports concluded that MIKC*-type and type I genes were probably of minor functional importance in plants. In comparison to type II genes, most type I genes are pseudogenes or functionally redundant^3, 34, 35^. Some reports address the role that MIKC*-type and type I MADS-box genes play in pollen development^36^, as well as embryo sac, endosperm and seed development and their contribution to postzygotic compatibility and reproductive isolation between species^37–40^. However, banana has unique characteristics. The diploids of *Musa acuminata* gave rise to seedless fruit after they became parthenocarpic and sterile^41^. Consequently, pollination and fertilization are of lesser importance in banana plants^42^. As such, it is possible that the MIKC*-type and type I MADS-box genes involved in pollination and fertilization have undergone a combination of subfunctionalization and neofunctionalization^43^. This was solidly demonstrated in this research by the result that 10 Mα/β and two MIKC* genes displayed high expression value during fruit development and ripening process in both varieties tested.

### Validation of the differentially expressed *MaMADSes* genes by qRT-PCR analysis

**Figure 6:**
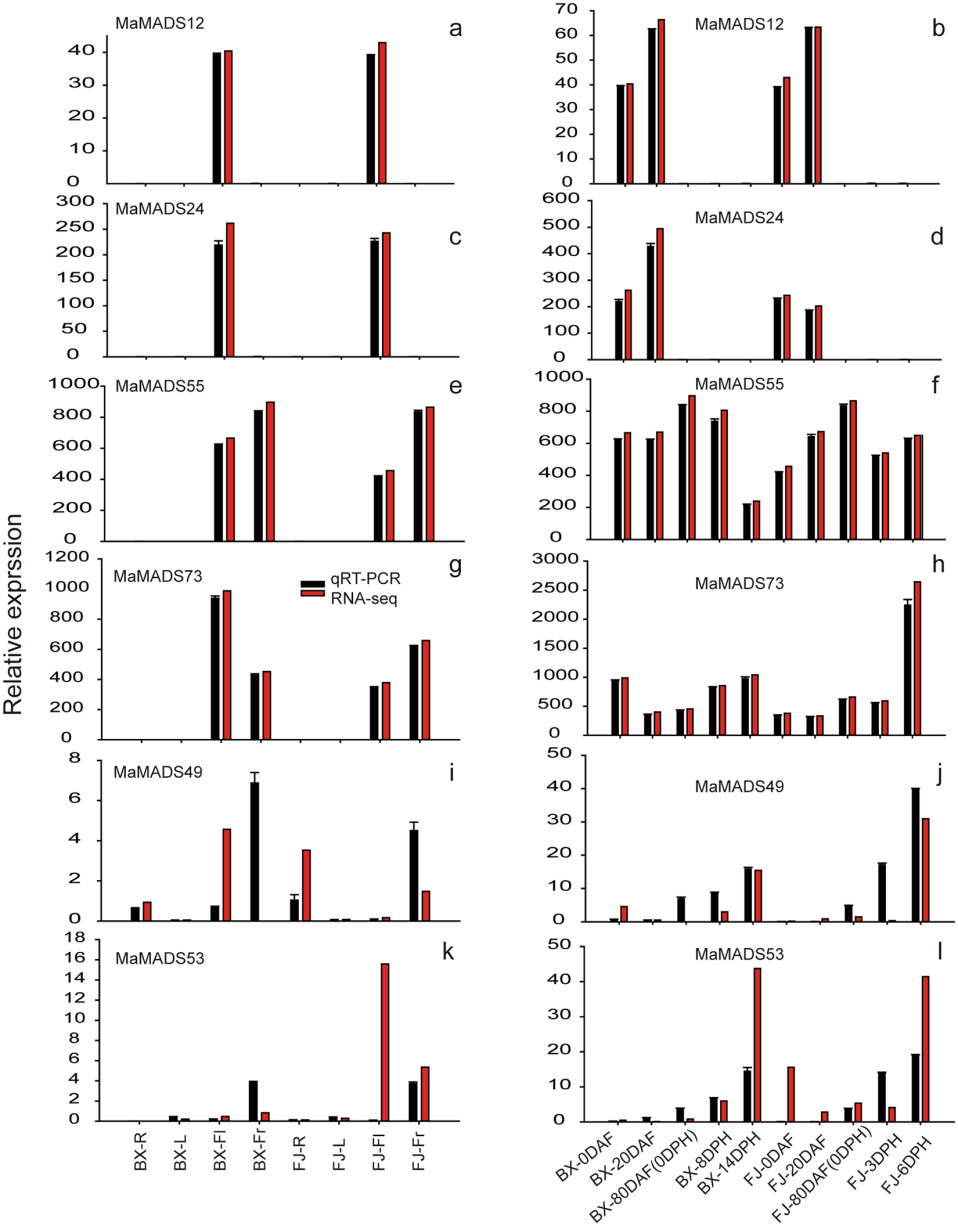
Relative expression of *MaMADSes* genes in BX and FJ by qRT-PCR. Expression patterns of *MaMADS12*, *24*, *55*, *73*, *49* and *53* in different organs (**a**,**c**,**e**,**g**,**i**,**k**). Expression patterns of *MaMADS12*, *24*, *55*, *73*, *49* and *53* in different stages of fruit development and ripening (**b**,**d**,**f**,**h**,**j**,**l**). Data are means ± SD of n = 3 biological replicates. When absent, the bars fall within the dimensions of the symbol.

According to the RNA-seq data, two BS subfamily genes *MaMADS12* and *24* characterized by highly expressed during banana fruit developmental processes, two SEP/AGL2 subfamily genes *MaMADS55* and 73 characterized by highly expressed during the fruit development and ripening processes and two genes of *MaMADS49* (Mα/β) and *53* (SEP/AGL2), were highly expressed during banana fruit ripening processes. These were selected for quantitative reverse transcriptase polymerase chain reaction (qRT-PCR) analysis to validate the RNA-seq data. After normalization, we found that all the selected *MaMADSes* genes, apart from *MaMADS49* and *53* exhibited the same trend with consistent results between the RNA-seq data and qRT-PCR data (Fig. 6). These results indicate that RNA-seq data are suitable for investigating the expression patterns of MADS genes in the two banana varieties.

### Interaction networks of preferentially expressed genes during the fruit development and ripening process

**Figure 7:**
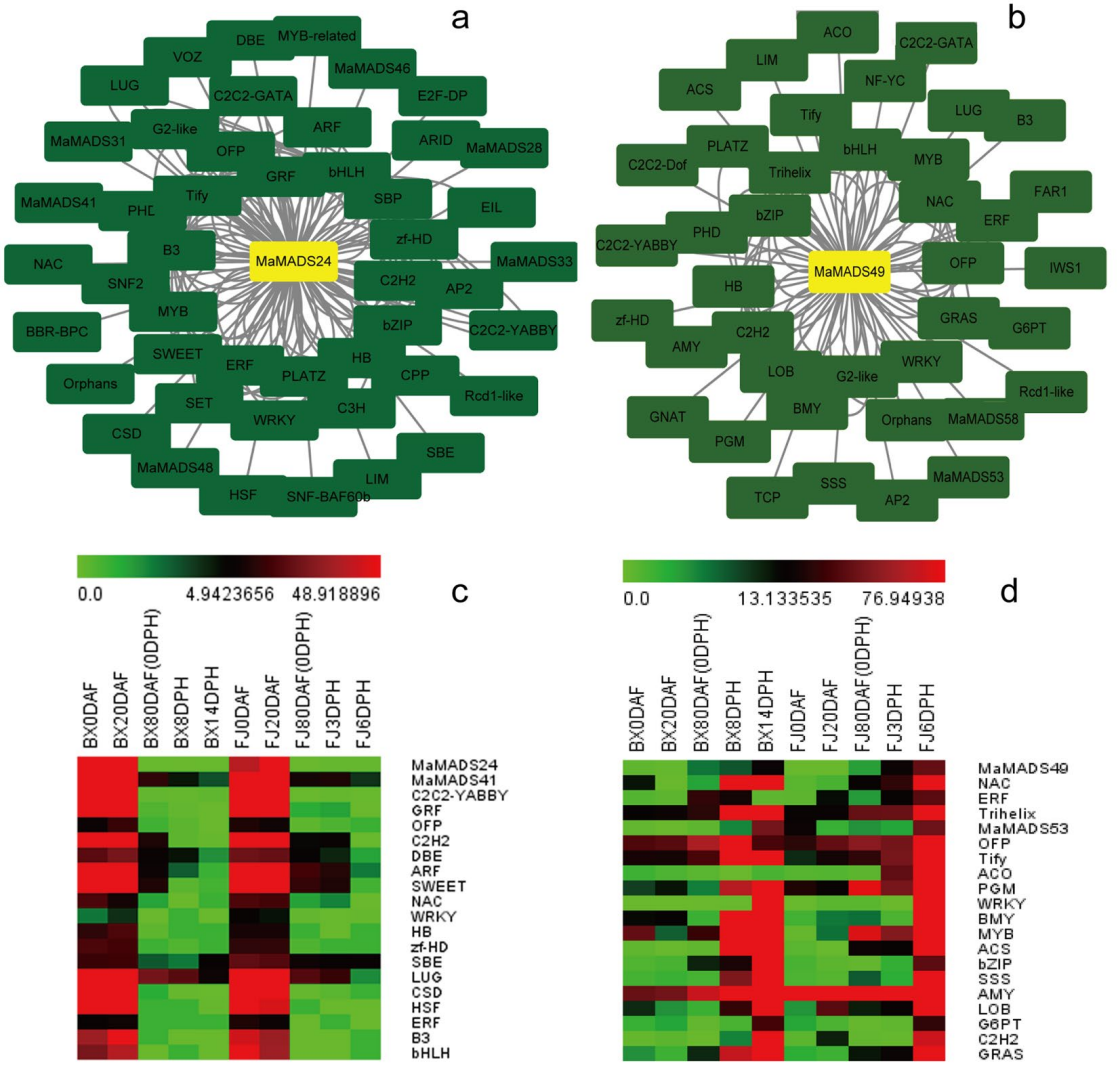
The interactive networks of MaMADS24 and 49 using Cytoscape and validation by qRT-PCR. (**a**) interaction network of MaMADS24 during fruit development process; (**b**) interaction network of MaMADS49 during fruit ripening process; (**c**) validation of the 19 genes interacted with MaMADS24; (**d**) validation of the 19 genes interacted with MaMADS49.

As MADS-box transcription factors typically meditate their function in biological processes through the interaction networks, studies on gene interaction networks are very useful for investigating the mechanism of gene function. In this study, *MaMADS24* and *49*, which were highly expressed during the fruit development stage and ripening stage, respectively, were selected to identify the potential protein interaction and co-expression networks using Cytoscape^44^. Two MaMADS-mediated networks were constructed and 47 and 40 interactive proteins for MaMADS24 and 49 were obtained, respectively (Fig. 7a and b; Supplementary Tables S4 and S5). MaMADS24 and 49 not only interacted with MaMADSes proteins, but also interacted with hormone-response proteins, ethylene signal transduction and biosynthesis- related proteins, starch biosynthesis and metabolism-related proteins. The 135 key proteins that interacted with MaMADS24 were divided into 42 classes, including bHLH (13), followed by MYB (8), GRF (8) and B3 (8); with single members for SWI.SNF-BAF60b, C2C2-YABBY, EIL, BBR-BPC, DBE, E2F-DP, etc. The 89 key proteins that interacted with MaMADS49 were divided into 39 classes including the most abundant TF family C2H2 (8), followed by the bZIP (7), NAC (6) and bHLH (6); with single members for ACO, C2C2-GATA, PGM, FAR1, LIM, ACS, etc. The full names of these interacted proteins with MaMADS24 and 49 were listed in Supplementary Table S8. Upon comparison of the proteins interacting with MaMADS24 and 49, 23 kinds of proteins including MaMADSes, bHLHs, MYB, B3, HB, Tify, C2H2, OFP, zf-HD, bZIP, ERF, PLATZ, WRKY, PHD, C2C2-GATA, G2-like, AP2, C2C2-YABBY, NAC, LIM, Rcd1-like, LUG and Orphans were found to be common, suggesting that these types of proteins may be fundamental for banana fruit development and ripening. Another 19 kinds of proteins including GRF, SBP, SWEET, ARF, C3H, SET, CPP, SNF2, SWI.SNF-BAF60b, EIL, BBR-BPC, DBE, E2F-DP, MYB-related, SBE, CSD, ARID, HSF and VOZ were found to be specifically interacted with MaMADS24, suggesting that these proteins may interact with MaMADS24 to participate banana fruit developmental process. On the other hand, 16 kinds of proteins including LOB, Trihelix, BMY, AMY, GRAS, ACO, PGM, FAR1, ACS, C2C2-Dof, IWS1, NF-YC, SSS, G6PT, TCP and GNAT were found to be specifically interacted with MaMADS49, suggesting that these proteins may interact with MaMADS49 to participate banana fruit ripening process.

By comparing the interactive networks of MaMADS24 and MaMADS49, two meaningful phenomena were observed. First, the main proteins interacting with MaMADS24 were divided into two classes. The one class comprised the plant hormone-response TFs participating in growth and development, such as GRF, SBP, ARF, C3H, SET, CPP, SNF2, SWI.SNF-BAF60b, EIL, E2F-DP, MYB-related, HSF and VOZ, which suggested that these proteins respond to plant hormones (indole-3-acetic acid, gibberellin, jasmonate, cytokine, abscisic acid and ethylene) to regulate banana flower and fruit development. The other constituted the key proteins of starch biosynthesis and transportation such as SBE, DBE and SWEET, suggesting their important roles in banana fruit starch biosynthesis, transportation and accumulation. Second, the main interactive proteins with MaMADS49 were divided into two classes. The one constituted the key enzyme of ethylene biosynthesis such as ACO and ACS, which was in accordance with the reports in tomato that RIPENING INHIBITOR (MADS-RIN) interacts directly with the promoters of genes involved in ethylene biosynthesis, *ACS2* and *ACS4* ^45, 46^. This result was consistent with the report in apple that a SEP-like *MADS9* was shown to act as a transcriptional activator of *ACS1* and *ACO1* promoter^47^. The other constituted the key proteins of starch converted to sugar such as AMY, BMY, PGM, SSS, G6PT, GNAT and NF-YC, suggesting their importance in banana fruit starch conversion and quality formation. These phenomena were highly consistent with the typical characteristics of banana fruit for its climacteric and starch conversion. This result was in accordance with Ireland *et al*.^47^, who reported that apple *MADS8*/*9* genes were found to control fruit ripening characters such as starch degradation and ethylene modulated ripening traits.

In order to further validate the protein interactions with MaMADS24 and MaMADS49, 19 predicted genes were selected to perform qRT-PCR. As shown in Fig. 7c and d, all the selected genes that interacted with MaMADS24 were highly expressed during the fruit development process. Conversely, all the selected genes that interacted with MaMADS49 were highly expressed during the fruit ripening process. Interestingly, *ACO* displayed higher expression levels during the fruit ripening process in FJ than in BX, suggesting an important role in the fruit ripening process. This result demonstrated that these predicted partners were consistent with *MaMADS24* and *MaMADS49* at the level of gene expression. Partially interacting proteins have been validated in the literature. Choudhury *et al*.^48^ discovered that an AGAMOUS MADS-box protein interacted with the CArG-box in banana flowers and fruit nuclear extracts in DNA-protein interaction assays. Recently, Liu *et al*.^19, 49^, using yeast two-hybrid screening, discovered that banana MADS-box (MuMADS1, also MaMADS5, closely linked with MaMADS36) interacted with MaOFP in 2 DPH banana fruit. The authors also discovered by means of a yeast one-hybrid assay that an AG-like MADS-box MaMADS7 (closely linked with MaMADS62) interacted with a *MaACO1* promoter.

In conclusion, 96 MADS-box genes were identified from the banana (Pahang) A genome, which could be divided into MIKC^c^, MIKC*, Mα/β and Mγ groups based on phylogeny and MIKC^c^ could be further divided into 11 subfamilies. The major genes that were highly expressed in fruit development and ripening belonged to SEP/AGL2, AG and AP1/FUL subfamilies, Several I-type, MIKC* and other subfamilies such as TM3/SOC1, BS, DEF/GLO and AGL17 MADS-box genes were highly expressed during banana fruit development and ripening, suggesting their previously unknown roles in these processes. Interactive network analysis indicated that MaMADS24 and 49 not only interacted with MaMADSes proteins themselves, but also interacted with other targets. Hormone response proteins and those involved in starch biosynthesis and transportation interacted with MaMADS24 during the fruit developmental process, while those involved in ethylene signal transduction, ethylene biosynthesis and starch metabolism interacted with MaMADS49 during the fruit ripening process. These results will advance our understanding of the roles of *MaMADSes* in the regulation of the signal transduction pathways of banana developmental and ripening processes, enabling improved breeding and genetic modification in agriculture.

## Methods

### Plant materials and treatments

Banana fruits at different stages of development (cultivars BX and FJ), including at 0 DAF, 20 DAF and 80 DAF (0 day post-harvest: 0 DPH), were obtained from the banana plantation of the Institute of Tropical Bioscience and Biotechnology banana plantation (Chengmai, Hainan, 20 N, 110E). Post-harvest banana hands at similar developmental stages were selected and allowed to ripen naturally. As ethylene production in variety FJ allows it to reach a fully yellow color faster than BX post-harvesting, the fruits of BX were obtained 8 DPH and 14 DPH, while those of FJ were obtained 3 DPH and 6 DPH^29, 50^. The samples were frozen in liquid nitrogen and stored at −80 °C until they were required for total RNA extraction for transcriptome analysis.

### Identification and evolutionary analyses

The whole MADS-box protein sequences of banana, Arabidopsis and rice were obtained from the Banana Genome Hub, released in January 2016 (http://banana-genome-hub.Southgreen.fr/download)^24^, RGAP (http://rice.plantbiology.msu.edu/) and TAIR (http://www.arabidopsis.org/) databases, respectively, respectively. To identify the banana MADS-box family genes, the local Hidden Markov Model-based searches (http://hmmer.wustl.edu/) were firstly built from known MADS-box to search the banana genome database^51^. Subsequently, BLAST searches were performed to check the predicted *MaMADSes* in the banana database using all Arabidopsis and rice *MADSes* as queries. Finally, all candidate protein sequences were further examined by the CDD (http://www.ncbi.nlm.nih.gov/cdd/) and PFAM (http://pfam.sanger.ac.uk/) databases. Then, multiple sequence alignments were used to confirm the conserved domains of predicted MaMADSes proteins. The full-length MADSes protein sequence alignments from banana, Arabidopsis and rice were aligned using Clustal X v.2.0. Relationships were assessed using a bootstrap neighbor-joining evolutionary tree with 1,000 bootstrap replicates, created using MEGA 5.0 software^52^.

### Protein properties and sequence analyses

The ExPASy proteomics server (http://expasy.org/) was employed to detect the molecular weight and isoelectric points of predicted MaMADSes proteins. Using the MEME program (http://meme.nbcr.net/meme/cgi-bin/meme.cgi), the conserved motifs in the full-length banana MADS proteins were identified with the following parameters: maximum number of motifs was 10 and the optimum width of motifs was set between six and 50. Subsequently, all identified motifs were annotated with the help of InterProScan (http://www.ebi.ac.uk/Tools/pfa/iprscan/). The gene structure display server program (http://gsds.Cbi.pku.Edu.cn/) was employed to identify the gene structures of banana *MaMADSes*.

### Transcriptome analysis

Banana fruits at different stages of development and ripening including 0 DAF, 20 DAF, 80 DAF (0 DPH), 8 DPH and 14 DPH for BX, and 3 DPH and 6 DPH for FJ, were collected for total RNA extraction using the plant RNeasy extraction kit (TIANGEN, Beijing, China) for transcriptome analysis. The sequencing was performed with an Illumina GAII following the manufacturer’s instructions. There were two replicates for each sample. The sequencing depth was 5.34X on average. Using the FASTX-toolkit, adapter sequences in the raw sequence reads were removed. After examining the sequence quality and removing low quality sequences by FastQC, clean reads were generated. Using Tophat v.2.0.10, clean reads were mapped to the DH-Pahang genome (*Musa acuminata*, A-genome, 2n = 22)^24^. The transcriptome was assembled using Cufflinks^53^. Gene expression levels were calculated as Reads per Kilobase of exon model per Million mapped reads (FPKM). DEGseq was used to identify differentially expressed genes^54^. RNAseq reads was deposited in NCBI-SRA database (accession number: PRJNA343716).

### Quantitative real-time PCR analysis

Transcriptional changes in the *MaMADSes* genes responding to fruit development and ripening and 38 selected genes interacted with MaMADS24 and 49 were determined by qRT-PCR analysis on a Stratagene Mx3000 P Real-Time PCR system using SYBR® Premix Ex Taq™ (TaKaRa, Japan). The PCR amplification conditions used for all reactions were implemented as follows: 10 min at 95 °C, followed by 40 cycles of 10 s at 95 °C, 15 s at 50 °C, and 30 s at 72 °C. The relative expression levels of the target genes were calculated using the 2−ΔΔCt method^55^. Reaction specificities for each primer pair were tested using qRT-PCR melting curve analysis, agarose gel electrophoresis and sequencing of PCR products (Supplementary Table S6). *MaRPS2* (HQ853246) and *MaUBQ2* (HQ853254) were used as the internal controls to normalize the expression of target genes in banana^56^. Each treated sample contained a corresponding regularly-watered control and each sample had three independent biological replicates. The treated and control plants at each time point were sampled for expression analysis. The relative expression levels of the *MaMADSes* genes in each treated time point were compared with that in each time point at normal conditions.

### Construction of the regulatory networks

Using Cytoscape software (v.3.4.0), we established the gene regulatory network diagram based on the results of the A genome database (http://banana-genome-hub.southgreen.fr/ download) and transcriptome analysis, we chose *MaMADS24*, which was specially expressed during the fruit development process, and *MaMADS49*, which was specially expressed during the fruit ripening process in BX and FJ as the “from node”, while the interactive proteins were selected as “to node direction”.

## Electronic supplementary material

Supplementary files
