# Supplementary material for: Genome-wide analysis of banana MADS-box family closely related to fruit development and ripening
Source: Sci Rep. 2017 Jun 14;7:3467. doi: 10.1038/s41598-017-03897-1 (PMC5471262; doi:10.1038/s41598-017-03897-1)
Supplement: Supplementary file 1 — Supplementary files [file 41598_2017_3897_MOESM1_ESM.pdf]

Genome-wide analysis of banana MADS-box family closely related to fruit development and ripening

Juhua Liu[1#], Jing Zhang[1#], Jianbin Zhang[1#], Hongxia Miao[1], Jingyi Wang[1], Pengzhao Gao[1], Wei Hu[1], Caihong Jia[1], Zhuo Wang[1], Biyu Xu[1*], Zhiqiang Jin[1, 2*]

1 Key Laboratory of Tropical Crop Biotechnology, Ministry of Agriculture; Institute of Tropical Bioscience and Biotechnology, Chinese Academy of Tropical Agricultural Sciences, 4 Xueyuan Road, 571101 Haikou, China;

2 Key Laboratory of Genetic Improvement of Bananas, Hainan Province; Haikou Experimental Station, Chinese Academy of Tropical Agricultural Sciences, 570102 Haikou, China.

*Corresponding author:

Biyu Xu (biyuxu@126.com);

Zhiqiang Jin (jinzhiqiang@itbb.org.cn)

# These authors contributed equally to this work.

Juhua Liu(juhua69@126.com)

Jing Zhang(zhangjing@itbb.org.cn)

Jianbin Zhang(zhangjianbin@itbb.org.cn)

Hongxia Miao(miaohongxia@itbb.org.cn)

Jingyi Wang(wangjingyi@itbb.org.cn)

Pengzhao Gao(gaopengzhao@163.com)

Wei Hu(huwei2013@itbb.org.cn)

Caihong Jia(jiacaihong@itbb.org.cn)

Zhuo Wang(wangzhuo@itbb.org.cn)

*Biyu Xu(xubiyu@itbb.org.cn)

*Zhiqiang Jin(jinzhiqiang@itbb.org.cn)

Table S1. Characteristics of *MaMADSes* in banana

Gene name	Gene ID	AA	MW(kDa)	PI	Group	Subfamily
MaMADS8	Ma01_t11930.1	226	25.8845	9.1	$MIKC^C$	DEF
MaMADS9	Ma01_t18380.1	212	24.1767	6.79	$MIKC^C$	TM3/SOC1
MaMADS10	Ma01_t18390.1	244	28.119	8.76	$MIKC^C$	SEP/AGL2
MaMADS11	Ma02_t02200.2	201	22.4623	6.49	I type	Mα/β
MaMADS12	Ma02_t04560.1	240	27.9796	6.18	$MIKC^C$	BS
MaMADS13	Ma02_t06310.3	213	24.45041	8.88	$MIKC^C$	TM3/SOC1
MaMADS14	Ma02_t06320.1	240	27.8546	7.77	$MIKC^C$	SEP/AGL2
MaMADS15	Ma02_t08180.2	240	27.4521	6.22	$MIKC^C$	SEP/AGL2
MaMADS16	Ma02_t12050.1	242	27.7908	9.16	$MIKC^C$	SEP/AGL2
MaMADS17	Ma02_t15490.1	246	28.2746	9.27	$MIKC^C$	SQUA/AP1
MaMADS18	Ma03_t02150.1	263	30.0615	9.18	$MIKC^C$	SQUA/AP1
MaMADS19	Ma03_t04320.1	337	37.7237	5.62	MIKC*	
MaMADS20	Ma03_t06740.1	225	25.2258	8.97	$MIKC^C$	STMADS11
MaMADS21	Ma03_t07260.1	244	28.0288	8.58	$MIKC^C$	AGL17
MaMADS22	Ma03_t17290.1	217	25.0338	8.7	$MIKC^C$	SEP/AGL2
MaMADS23	Ma03_t21090.1	240	27.4144	8.97	$MIKC^C$	AGL17
MaMADS24	Ma03_t24850.1	240	28.076	6.67	$MIKC^C$	BS
MaMADS25	Ma03_t26480.1	244	28.055	8.95	$MIKC^C$	SQUA/AP1
MaMADS26	Ma03_t26910.1	228	25.4833	9.6	I type	Mα/β
MaMADS27	Ma03_t31640.1	175	20.4826	9.67	$MIKC^C$	SQUA/AP1
MaMADS28	Ma04_t14430.1	240	27.2821	8.58	$MIKC^C$	SEP/AGL2
MaMADS29	Ma04_t20900.1	242	27.6505	9.28	$MIKC^C$	SEP/AGL2
MaMADS30	Ma04_t23400.1	255	28.8641	9.31	I type	Mα/β
MaMADS31	Ma04_t30020.1	248	28.3744	9.28	$MIKC^C$	SQUA/AP1
MaMADS32	Ma04_t36630.1	235	26.7724	8.99	$MIKC^C$	SEP/AGL2
MaMADS33	Ma05_t01150.1	211	24.5402	9.13	$MIKC^C$	GLO
MaMADS34	Ma05_t06360.1	230	26.9024	8.85	$MIKC^C$	AG
MaMADS35	Ma05_t10000.1	216	24.4689	6.13	$MIKC^C$	TM3/SOC1
MaMADS36	Ma05_t18560.1	235	26.9614	8.82	$MIKC^C$	AG
MaMADS37	Ma05_t26810.1	236	27.2081	9.3	$MIKC^C$	AGL17
MaMADS38	Ma05_t28910.1	225	24.4632	9.22	I type	Mα/β
MaMADS39	Ma06_t01760.1	194	22.6352	9.24	$MIKC^C$	SQUA/AP1
MaMADS40	Ma06_t07660.1	217	24.8	8.74	$MIKC^C$	SEP/AGL2
MaMADS41	Ma06_t16260.1	222	25.4309	9.4	$MIKC^C$	AG
MaMADS42	Ma06_t20000.1	211	24.5602	7.06	$MIKC^C$	GLO
MaMADS43	Ma07_t00440.1	250	28.4877	9.18	$MIKC^C$	SQUA/AP1
MaMADS44	Ma07_t03020.1	229	25.7789	6.75	$MIKC^C$	AGL12
MaMADS45	Ma07_t12420.1	240	27.7459	9.52	$MIKC^C$	AGL17
MaMADS46	Ma07_t25120.1	239	27.6923	9.07	$MIKC^C$	AG
MaMADS47	Ma07_t28230.1	241	27.6081	7.63	$MIKC^C$	SEP/AGL2
MaMADS48	Ma08_t04270.1	245	27.9627	7.74	$MIKC^C$	SQUA/AP1
MaMADS49	Ma08_t04740.1	215	24.2188	8.9	I type	Mα/β
MaMADS50	Ma08_t07050.1	236	25.7545	9.46	I type	Mα/β
MaMADS51	Ma08_t08940.2	221	25.0473	6.78	I type	Mα/β
MaMADS52	Ma08_t12030.1	210	23.9623	8.58	$MIKC^C$	SQUA/AP1

MaMADS53	Ma08_t28680.1	185	21.5997	9.08	$MIKC^C$	SEP/AGL2
MaMADS54	Ma08_t28790.1	224	25.7957	9.3	I type	Mα/β
MaMADS55	Ma09_t01340.1	243	27.6877	8.81	$MIKC^C$	SEP/AGL2
MaMADS56	Ma09_t05610.1	197	22.646	8.74	$MIKC^C$	OsMADS32-like
MaMADS57	Ma09_t14590.1	224	25.1629	8.29	$MIKC^C$	STMADS11
MaMADS58	Ma09_t14960.1	249	28.7178	9.31	$MIKC^C$	AGL17
MaMADS59	Ma09_t21260.1	225	26.0926	9.39	$MIKC^C$	DEF
MaMADS60	Ma09_t29900.1	245	27.8366	9.08	$MIKC^C$	SEP/AGL2
MaMADS61	Ma10_t07670.2	212	24.3664	8.99	$MIKC^C$	TM3/SOC1
MaMADS62	Ma10_t11270.1	234	27.1294	8.81	$MIKC^C$	AG
MaMADS63	Ma10_t14260.1	222	25.6272	9.45	$MIKC^C$	AG
MaMADS64	Ma10_t18550.1	238	27.5847	9.3	$MIKC^C$	SQUA/AP1
MaMADS65	Ma10_t19310.2	228	26.0306	5.85	$MIKC^C$	STMADS11
MaMADS66	Ma10_t21430.1	232	26.9235	9.5	$MIKC^C$	AG
MaMADS67	Ma10_t29960.2	293	33.3211	8.61	$MIKC^C$	AGL17
MaMADS68	Ma11_t02670.1	212	24.0505	9.06	$MIKC^C$	TM3/SOC1
MaMADS69	Ma11_t02680.3	214	24.2238	8.99	$MIKC^C$	TM3/SOC1
MaMADS70	Ma11_t07440.1	208	23.9525	9.22	$MIKC^C$	TM3/SOC1
MaMADS71	Ma11_t14170.1	207	23.0317	9.03	I type	Mα/β
MaMADS72	Ma11_t17800.1	224	25.8518	6.26	$MIKC^C$	AGL12
MaMADS73	Ma11_t17960.1	235	26.9206	8.93	$MIKC^C$	SEP/AGL2
MaMADS74	Ma11_t19830.1	215	24.8552	8.91	$MIKC^C$	TM3/SOC1
MaMADS75	Ma11_t19840.1	250	28.6198	8.19	I type	Mα/β
MaMADS76	Ma02_t03680.1	341	38.575	5.42	MIKC*	
MaMADS77	Ma03_t25430.1	348	39.2279	6.1	MIKC*	
MaMADS78	Ma02_t13660.1	146	16.7333	9.19	$MIKC^C$	TM3/SOC1
MaMADS79	Ma04_t05410.1	174	19.1563	10.7	I type	Mα/β
MaMADS80	Ma05_t01930.1	527	56.5764	5.97	I type	Mα/β
MaMADS81	Ma03_t08420.1	230	26.4645	8.7	I type	Mγ
MaMADS82	Ma11_t16180.1	253	28.6801	8.35	I type	Mα/β
MaMADS83	Ma09_t21270.1	102	11.7295	9.3	$MIKC^C$	DEF
MaMADS84	Ma09_t06890.1	191	21.4086	9.63	I type	Mγ
MaMADS85	Ma11_t12730.1	250	28.2769	5.92	MIKC*	
MaMADS86	Ma02_t20230.1	232	26.4794	8.64	I type	Mγ
MaMADS87	Ma08_t08930.1	126	14.3067	10.8	I type	Mα/β
MaMADS88	Ma03_t05320.1	221	25.6018	9.11	I type	Mγ
MaMADS89	Ma03_t25760.1	232	26.7871	8.9	I type	Mγ
MaMADS90	Ma11_t14180.1	159	17.7875	9.97	I type	Mα/β
MaMADS91	Ma08_t11420.1	122	13.9898	6.59	I type	Mα/β
MaMADS92	Ma08_t06010.1	245	28.0072	9.21	I type	Mγ
MaMADS93	Ma11_t24130.1	96	10.4	8.77	I type	Mα/β
MaMADS94	Ma04_t34810.1	246	27.0987	6.19	I type	Mα/β
MaMADS95	Ma05_t22690.1	63	7.2074	10.1	$MIKC^C$	TM3/SOC1
MaMADS96	Ma05_t01940.1	347	37.5349	8.51	I type	Mα/β
MaMADS97	Ma06_t26090.1	267	30.3155	5.48	I type	Mγ
MaMADS98	Ma01_t20740.1	150	16.4724	9.59	I type	Mα/β
MaMADS99	Ma04_t11870.1	152	16.8837	10.3	I type	Mα/β

MaMADS100	Ma05_t27740.1	234	25.833	7.46	I type	Mα/β
MaMADS101	Ma05_t01890.1	244	26.8085	9.04	I type	Mα/β
MaMADS102	Ma09_t12630.1	342	38.5308	5.75	MIKC*	
MaMADS103	Ma10_t05280.1	233	27.1685	8.54	I type	Mγ

Note: The number designation was based on the order of the multiple sequence alignments and seven previously functionally characterized genes, *MaMADS1 -MaMADS7* (Elitzur et al., 2010; Liu et al., 2015).

Table S2. The expression data of *MaMADSes* in different tissues of BX and FJ varieties.

Gene	BX-R	BX-L	BX-Fl	BX-Fr	FJ-R	FJ-L	FJ-Fl	FJ-Fr
MaMADS8	0.048076199	0	0.627854177	0	0	0	2.345127407	0.425658003
MaMADS9	20.48057439	65.69873911	16.38976326	0.488270688	45.00533851	226.82355	42.87181686	0.113275707
MaMADS10	0	0	2.639222001	0.473250975	0	0.071218319	1.061838042	3.077400226
MaMADS11	25.87361264	47.00673812	35.10922903	169.5419974	26.14308436	12.86235972	12.88096523	26.09378004
MaMADS12	0.047633078	0	40.4122677	0.050588758	0	0	42.96253735	0
MaMADS13	17.7705645	17.79841365	16.18561709	3.696016416	16.7535144	10.94300789	12.86501592	2.453161647
MaMADS14	0	0.178725994	9.802938542	7.324505126	0	0.241642127	28.65087423	10.06368051
MaMADS15	0.557498807	5.455535717	8.415425212	25.39620131	0.342948247	0.846208809	21.06834259	27.06138709
MaMADS16	0	1.038690903	3.744472881	3.453330018	0.292188606	0.239805825	118.2709726	12.73232635
MaMADS17	0.185903995	0.086232682	95.39209178	482.7638987	0.239796924	0.070791705	119.2471425	443.260179
MaMADS18	0.254464418	0.811586274	29.81135523	31.4029155	4.319832414	22.63545124	42.33898693	70.03120611
MaMADS19	NA	NA	NA	NA	NA	NA	NA	NA
MaMADS20	49.34289626	66.15023583	6.499116435	6.259233915	43.23867639	31.78147894	11.21845387	23.08644043
MaMADS21	4.929792757	1.249165661	2.818742931	1.224231053	4.942126995	0.237999504	1.136494399	0.074207725
MaMADS22	0	0.024426001	0	0	0.053999959	0	6.552412087	0.35853125
MaMADS23	4.420581283	0.044845406	0	0	3.773965505	0	0	0
MaMADS24	0.11790785	0	261.6858711	0.329339491	0.024423218	0	242.6818073	0.125261507
MaMADS25	1.417847359	0	1.990207333	0.600431123	0.651344705	0.21395751	6.767474449	5.515140242
MaMADS26	1.292566234	1.405511722	6.438207662	11.83709561	1.729820649	0.686881366	7.028501386	9.145910022
MaMADS27	0	0.06140763	0.342578608	0.695879465	0.066886313	0.06619805	0.75749657	4.186060297
MaMADS28	3.004192571	0	2.024429278	0.101690084	1.127901872	0.870572946	97.91622108	13.78555066
MaMADS29	0	0	42.69626122	26.38020984	0	0	72.16947721	30.45998141
MaMADS30	1.618234656	0.189979854	0.406794393	0.095490413	2.978993704	2.731031508	0.543771503	0.046902853
MaMADS31	7.955499886	0.042770025	110.9123365	129.0970545	0.900575129	0	103.542641	30.02444802
MaMADS32	0.07296338	0.091591041	42.24278084	61.12028039	0	0	80.60023656	64.2630544
MaMADS33	2.617600682	0	51.52493171	0.201281211	3.677853766	1.236573321	104.0473695	1.365452705
MaMADS34	0.289590775	0.023051377	12.05320709	0	1.048258803	0	22.87360433	2.037001642
MaMADS35	7.200020001	2.594024459	1.262774638	0.056183827	5.035628195	1.181619849	0.306562978	0

MaMADS36	0.216490669	0.364690364	716.54396	452.1392049	0.37585134	0.296522317	348.0997989	115.3499513
MaMADS37	2.317263962	0.181075772	0.069402132	0.956378633	5.704653208	0.123094813	0.178299139	0.381169366
MaMADS38	NA	NA	NA	NA	NA	NA	NA	NA
MaMADS39	0.259105248	0.659419831	3.681868283	1.632870448	0.121159891	0.059652951	19.30772811	0.586999309
MaMADS40	0.131646465	0.097704002	165.9794647	287.6751158	0.215999836	0	165.9335507	230.0234821
MaMADS41	1.435378297	0.023878332	63.11324203	13.90683732	0.317103781	0	87.91892641	9.195649044
MaMADS42	3.242012245	2.455383835	10.98802838	0.982021729	4.433301072	5.274198852	24.1489475	2.363029866
MaMADS43	8.208872755	0	11.66051318	1.487390475	7.716220257	0	27.68015916	7.714030295
MaMADS44	4.489987989	0	0	0	2.079148173	0	0	0
MaMADS45	0.393132723	0.156303291	0.318328686	0.152535125	1.324651875	0.048420698	0.100319153	0.074733177
MaMADS46	0.488074396	0	1091.182496	27.88041079	0.418123385	0	845.9317849	20.42690333
MaMADS47	0.07115437	0.066663682	13.31661776	86.22218905	0	0	23.57718529	31.15385713
MaMADS48	0.750404872	4.318626904	39.07544327	10.98157603	1.128903848	3.172372198	33.47774278	9.195132643
MaMADS49	0.93565784	0.049304334	4.568108184	0	3.526473265	0.080780038	0.168243839	1.478087362
MaMADS50	0.093290006	0	3.191604077	0	0.074679684	0	0.995541293	0
MaMADS51	26.6973003	41.48413195	34.46478383	18.84561987	34.21668545	41.17248536	54.43646354	9.375608482
MaMADS52	5.515937915	7.137091635	1.896133772	0	10.38748633	20.79156466	2.094521427	0.227892199
MaMADS53	0	0.202946756	0.471189276	0.823996424	0.126801508	0.281825752	15.58271898	5.350449343
MaMADS54	2.822245374	1.123541805	8.769549063	10.35384463	0.0786626	0.698598418	0.861363837	0.080803967
MaMADS55	0.805923954	0.131586947	665.4472226	896.0832764	0.291496419	0.095650724	456.2401422	863.9829501
MaMADS56	0.028988818	0.135264379	0.664389153	0	0.119531703	0.147059982	0.672721836	0
MaMADS57	36.92518444	98.72757751	0.048717255	0	79.77690841	136.7178703	0.72534094	0.561593471
MaMADS58	3.819245729	2.68076281	8.066729929	1.860826159	4.486672465	2.027360275	6.341881497	2.290205022
MaMADS59	0.074939028	1.185409175	59.1960118	11.00250224	0.078132684	2.963241698	37.59361476	33.860148
MaMADS60	0.347685012	0	338.561753	798.1921432	0.26469852	0	355.6884121	824.476712
MaMADS61	7.647603908	0	0.565729698	0	6.683281313	0	0.425543877	0.113808405
MaMADS62	0.048849241	0.182616843	96.54990004	25.67383026	0.251866987	0.198154742	122.0402094	26.41131412
MaMADS63	6.768877307	17.51415331	13.36848413	6.295690976	0.740707444	0.208983979	112.2990525	77.38610025
MaMADS64	2.061268253	0.13467037	46.62327136	72.83481754	5.562360206	0.2435867	43.09763148	44.02017974
MaMADS65	16.71569401	48.79540625	0	0	45.54483006	145.9778097	0	0

MaMADS66	0.142944918	0.046385163	186.6245378	159.8522372	0.227708826	0.17489357	105.4180954	168.9648034
MaMADS67	6.605975025	0.091096419	1.21161146	0.396896814	3.093624785	0.138543096	3.395934088	0.904088798
MaMADS68	26.41708486	27.77238152	15.5670752	1.842084598	18.66043685	21.41673457	6.450021571	4.333994344
MaMADS69	1.769282006	4.796753706	0	0	0.933867076	1.734339593	0.225461881	0
MaMADS70	9.629314898	1.26051025	59.23839872	21.28029982	9.326516492	1.39364316	17.17070687	9.813254214
MaMADS71	2.313286253	5.983715302	14.07104012	3.859465464	1.98837223	5.265558341	15.76876212	3.279553856
MaMADS72	22.37273453	1.003455774	1.681216143	0.57197449	20.00075816	5.669960107	1.235775962	2.923774576
MaMADS73	0.216490669	0	988.1083635	452.3773322	0.626128658	0.098579029	378.7416972	657.9277133
MaMADS74	4.96141736	0.247984695	1.29413864	0.141109844	2.869239171	0.539219932	0.308679432	0.279518732
MaMADS75	7.976811917	11.56429303	0.633103132	0	1.788763322	0.464325223	0.168834138	0
MaMADS76	0.738677242	3.799305634	4.936009376	2.364198652	1.847653617	4.377669291	8.385059132	5.378773406
MaMADS77	1.115329306	2.368444011	3.910381854	0.982924288	1.216540249	1.218419704	1.68136014	0.724930441
MaMADS78	2.51926358	8.009282864	4.661002805	4.881706642	1.04385624	2.10038756	2.345883138	1.109642466
MaMADS79	1.194139591	14.22369465	3.820753502	1.644608469	0.03363426	5.092612313	0.138153805	0.103566631
MaMADS80	3.087794165	1.146934196	1.069232605	2.261871463	2.472752873	0.750104178	1.077510485	1.792779797
MaMADS81	0	0	2.325411493	1.909125768	0	0.050516833	0.497828878	0
MaMADS82	13.33935243	9.70741085	1.769062589	0.21599806	6.51244607	3.78419851	4.883878269	0.687903333
MaMADS83	0	0	1.596801843	0.118367869	0	0	0.821984298	0.879813606
MaMADS84	0.056840089	0.33609606	0.114310895	0.031749715	0.061740561	0.27292218	0.189431185	0.0631281
MaMADS85	0.086958543	0.106387824	0.15287279	0.146704102	0.04722784	0.046343926	0.072511764	0.072433835
MaMADS86	0.650543219	0.114945696	0.070647077	0.052855878	1.343284213	0.274900924	0.077984269	0.026009861
MaMADS87	0	0.294118739	0.560672153	1.350796619	0.185709296	0.642902795	0.190369417	0.237700969
MaMADS88	0.024579498	0	0.148070648	0	0.213218713	0.078847218	0.327122081	0.135470984
MaMADS89	0.023419093	0	0.141080188	0	0.076138126	0	0	0
MaMADS90	0.035873662	0	0.034293269	0	0	0	0	0
MaMADS91	0	0.219027266	0.04460913	0	0	0	0	0
MaMADS92	NA	NA	NA	NA	NA	NA	NA	NA
MaMADS93	0.695481892	0	0	0	0.121784427	0	0	0
MaMADS94	0.137135387	0.086232682	0	0	0	0	0	0
MaMADS95	0.529256882	0.5848763	0	0	1.751257893	4.186737114	0	0

MaMADS96	0.128694648	0.061886444	0	0	0	0	0.104340664	0
MaMADS97	0.020360629	0.201047416	0	0	0.505754816	2.044215338	0.406686155	0.112430189
MaMADS98	0	0	0	0	0.078504555	0	0	0
MaMADS99	0.108843679	0	0	0	0.038739176	0.076028271	1.266120971	0.039609788
MaMADS100	0	0.524182961	0	0	0.050268466	0.297705257	0	0
MaMADS101	NA	NA	NA	NA	NA	NA	NA	NA
MaMADS102	NA	NA	NA	NA	NA	NA	NA	NA
MaMADS103	NA	NA	NA	NA	NA	NA	NA	NA

Table S3. The expression data of *MaMADSes* in different fruit development and ripening stages in BX and FJ varieties.

Gene	BX0DAF	BX20DAF	BX80DAF(0DP	BX8DPH	BX14DPH	FJ0DAF	FJ20DAF	FJ80DAF(0DI	FJ3DPH	FJ6DPH
MaMADS8	0.627854177	0.719218763	0	0	0	2.345127407	0.922531963	0.425658003	0.051814641	0
MaMADS9	16.38976326	24.58257687	0.488270688	0	0.051478026	42.87181686	31.72220301	0.113275707	0.276504025	0
MaMADS10	2.639222001	3.332177984	0.473250975	0.022791069	0	1.061838042	1.030961762	3.077400226	1.394502412	0.171646329
MaMADS11	35.10922903	34.85220991	169.5419974	31.53830802	18.23672873	12.88096523	13.96789214	26.09378004	21.97830786	6.233537482
MaMADS12	40.4122677	66.36778824	0.050588758	0	0	42.96253735	63.35661574	0	0	0.050640158
MaMADS13	16.18561709	17.04451221	3.696016416	2.132069927	1.782077209	12.86501592	24.38358876	2.453161647	3.08169273	2.657365263
MaMADS14	9.802938542	13.09080465	7.324505126	12.98776659	6.110516645	28.65087423	3.067195208	10.06368051	4.177779321	2.246475437
MaMADS15	8.415425212	5.354226186	25.39620131	14.02017231	0.339164871	21.06834259	15.07954672	27.06138709	10.7746506	6.314166334
MaMADS16	3.744472881	11.42311422	3.453330018	67.7265201	4.680525208	118.2709726	19.3665953	12.73232635	2.205040567	18.54182986
MaMADS17	95.39209178	76.0501515	482.7638987	228.9667034	252.1230115	119.2471425	113.7089982	443.260179	244.0115235	318.4204635
MaMADS18	29.81135523	37.15739632	31.4029155	23.92366261	21.76666105	42.33898693	65.51927297	70.03120611	60.22105966	46.57706095
MaMADS20	6.499116435	5.605265596	6.259233915	5.807421946	5.150224128	11.21845387	17.6082833	23.08644043	22.61386612	6.11808909
MaMADS21	2.818742931	2.274936681	1.224231053	0.567264727	0.577407863	1.136494399	0.402300363	0.074207725	0.072186756	0.048013047
MaMADS22	0	0	0	0.025613816	0	6.552412087	0	0.35853125	0.027042439	0
MaMADS24	261.6858711	494.2514864	0.329339491	0.184176126	0.089618384	242.6818073	202.2680269	0.125261507	0.317586069	0.07504513
MaMADS25	1.990207333	1.263662392	0.600431123	0.204153468	0.555369056	6.767474449	2.187977802	5.515140242	4.469979519	3.767217579
MaMADS26	6.438207662	15.75700867	11.83709561	9.78808155	9.477745243	7.028501386	6.053268059	9.145910022	6.402507867	9.565929971
MaMADS27	0.342578608	0.254354733	0.695879465	0.819770575	0.523898525	0.75749657	0.349639214	4.186060297	1.304466747	7.583824916
MaMADS28	2.024429278	3.109729819	0.101690084	0	0	97.91622108	102.6389192	13.78555066	8.45423527	0.640019911
MaMADS29	42.69626122	93.67394476	26.38020984	31.15859812	4.097341534	72.16947721	62.08667966	30.45998141	15.5310314	10.33664988
MaMADS30	0.406794393	0.02185861	0.095490413	0	0.042831327	0.543771503	0.072125928	0.046902853	0	0.091899973
MaMADS31	110.9123365	87.87293285	129.0970545	6.54655364	3.253337676	103.542641	95.66565928	30.02444802	23.69448119	2.826527659
MaMADS32	42.24278084	51.92667703	61.12028039	131.4683663	10.39512971	80.60023656	58.49512286	64.2630544	36.30261352	25.45158209
MaMADS33	51.52493171	79.98918278	0.201281211	0.184147838	1.233870419	104.0473695	113.6352898	1.365452705	1.944388403	14.56352071
MaMADS34	12.05320709	5.509989994	0	0.024172346	0	22.87360433	12.05249154	2.037001642	0.739230478	0.337682088
MaMADS35	1.262774638	0.467084166	0.056183827	0.178813984	3.842173577	0.306562978	0	0	0.054334118	0
MaMADS36	716.54396	579.927714	452.1392049	83.45445265	45.40587746	348.0997989	666.5398802	115.3499513	100.4485016	69.45788643
MaMADS37	0.069402132	0.21427889	0.956378633	0.281725887	0	0.178299139	0.077908175	0.381169366	0.198754658	0.051494844
MaMADS39	3.681868283	2.338472938	1.632870448	0.427581777	0.055379566	19.30772811	1.453855388	0.586999309	0.151087019	0.15194119
MaMADS40	165.9794647	169.3942269	287.6751158	93.23910277	121.1814405	165.9335507	230.5402263	230.0234821	144.7646536	165.6119132
MaMADS41	63.11324203	78.85189971	13.90683732	4.515593346	3.36482013	87.91892641	97.40970546	9.195649044	10.58825478	3.919960516
MaMADS42	10.98802838	14.17700927	0.982021729	0.419856603	1.10300411	24.1489475	38.23941776	2.363029866	2.944188178	3.299030999

MaMADS43	11.66051318	8.687178924	1.487390475	0.44228469	0.280316349	27.68015916	21.08370792	7.714030295	10.18045985	12.98079792
MaMADS44	0.318328686	0.163408958	0.152535125	0.045945813	0.157864149	0.100319153	0.07688788	0.074733177	0.14647328	0.172665017
MaMADS45	1091.182496	1142.540422	27.88041079	4.495039196	3.580237355	845.9317849	660.9713861	20.42690333	23.72431224	89.82319815
MaMADS46	13.31661776	17.28538993	86.22218905	50.70786168	19.405315	23.57718529	30.80533221	31.15385713	18.22514417	15.37081584
MaMADS47	39.07544327	34.56230242	10.98157603	9.44483284	11.32056478	33.47774278	35.80502445	9.195132643	8.736331392	7.729745882
MaMADS48	4.568108184	0.522036121	0	2.9364915	15.41796871	0.168243839	0.884791072	1.478087362	0.299625736	30.95867087
MaMADS49	3.191604077	4.132341879	0	0	0.068697996	0.995541293	1.35091692	0	0.04974896	0.074450611
MaMADS50	34.46478383	41.3280911	18.84561987	2.605390558	0.83180625	54.43646354	78.08026718	9.375608482	39.25539665	2.750040433
MaMADS51	1.896133772	2.266896348	0	0	0.077556053	2.094521427	0.584374873	0.227892199	0.558317558	0.027874873
MaMADS52	0.471189276	0.121095895	0.823996424	5.945571615	43.75188377	15.58271898	2.814714455	5.350449343	4.116495838	41.4244861
MaMADS53	8.769549063	7.951547455	10.35384463	8.006392847	18.42155514	0.861363837	0.246189834	0.080803967	0.052275215	0.29342589
MaMADS54	665.4472226	669.2545904	896.0832764	806.0757665	238.507889	456.2401422	672.3237835	863.9829501	539.4570432	649.3846483
MaMADS55	0.664389153	1.703865539	0	0	0	0.672721836	0.279761175	0	0	0
MaMADS56	0.048717255	0.075235699	0	0	0	0.72534094	0	0.561593471	0.575725992	0.052280873
MaMADS57	8.066729929	8.061564766	1.860826159	0.956249595	2.047790127	6.341881497	4.607913517	2.290205022	2.802252944	23.09699791
MaMADS58	59.1960118	89.8173837	11.00250224	6.372543855	1.491918742	37.59361476	26.5205828	33.860148	11.46470878	18.33117003
MaMADS59	338.561753	398.8576781	798.1921432	572.6052741	751.6122762	355.6884121	545.48536	824.476712	653.9443096	777.5436812
MaMADS60	0.565729698	0.211161329	0	0	0	0.425543877	0.086995207	0.113808405	0.027677239	0
MaMADS61	96.54990004	116.8072138	25.67383026	19.52014809	2.849055654	122.0402094	146.2405414	26.41131412	23.30150435	6.659021877
MaMADS62	13.36848413	18.80580041	6.295690976	5.708998331	0.488350118	112.2990525	93.11857517	77.38610025	65.71956024	9.442520632
MaMADS63	46.62327136	40.37769307	72.83481754	34.68086417	21.03464453	43.09763148	47.40661495	44.02017974	33.33132575	32.88583611
MaMADS64	186.6245378	127.0671244	159.8522372	141.8172608	54.74153954	105.4180954	87.3288916	168.9648034	90.46851881	92.45327276
MaMADS67	1.21161146	0.574826814	0.396896814	0.265251702	0.185066623	3.395934088	6.201571561	0.904088798	1.161647893	0.243815927
MaMADS68	15.5670752	5.918788971	1.842084598	1.305419713	0.405596817	6.450021571	5.641416673	4.333994344	5.834328857	0.9391894
MaMADS70	59.23839872	51.17351911	21.28029982	1.967529845	0.600154105	17.17070687	26.46235566	9.813254214	19.32685488	2.60942734
MaMADS71	14.07104012	16.64466667	3.859465464	3.182749316	0.731639613	15.76876212	14.09332754	3.279553856	5.123407324	0.810838866
MaMADS72	1.681216143	1.324997502	0.57197449	0.396229441	0	1.235775962	0.57473513	2.923774576	3.139815501	1.378906326
MaMADS73	988.1083635	399.7418599	452.3773322	855.676471	1038.233258	378.7416972	333.5749542	657.9277133	591.8180076	2643.641134
MaMADS74	1.29413864	1.147047226	0.141109844	0.051701962	0	0.308679432	0.228308635	0.279518732	0.408598592	0.027229622
MaMADS75	0.633103132	1.32459232	0	0	0.043023965	0.168834138	0.048954494	0	0	0
MaMADS76	4.936009376	4.892407661	2.364198652	1.089890332	4.691891396	8.385059132	8.394909275	5.378773406	4.494202786	2.557287358
MaMADS77	3.910381854	4.969442423	0.982924288	0.493677259	5.278049824	1.68136014	1.88890022	0.724930441	0.455424518	0.754789909
MaMADS78	4.661002805	7.495988001	4.881706642	0.719462826	18.29191381	2.345883138	3.35250302	1.109642466	0.640979577	0.529143004
MaMADS79	3.820753502	3.991142054	1.644608469	1.49315979	6.27615737	0.138153805	0.21120769	0.103566631	0.269170594	1.241434602

MaMADS80	1.069232605	1.397274826	2.261871463	2.915488568	0.83524207	1.077510485	1.446034537	1.792779797	3.200502268	1.194697462
MaMADS81	2.325411493	1.65577212	1.909125768	0	0	0.497828878	1.467218112	0	0.101958558	0
MaMADS82	1.769062589	1.815654202	0.21599806	0.043594256	0.279616617	4.883878269	1.116148822	0.687903333	2.063183639	0.069467696
MaMADS83	1.596801843	2.082219573	0.118367869	0	0	0.821984298	0.419453854	0.879813606	0.28617727	0.175591032
MaMADS84	0.114310895	0.262669516	0.031749715	0	0	0.189431185	0.192335808	0.0631281	0.122520036	0
MaMADS85	0.15287279	0.179472801	0.146704102	0	0.043354253	0.072511764	0.049085453	0.072433835	0.164352479	0.215287203
MaMADS86	0.070647077	0.048636173	0.052855878	0	0.023529656	0.077984269	0.158773462	0.026009861	0.050480358	0
MaMADS87	0.560672153	0.574459713	1.350796619	0.350245112	0	0.190369417	0.097011407	0.237700969	0.417548707	0.048048339
MaMADS88	0.148070648	0.051046073	0	0	0.464360621	0.327122081	0.05534945	0.135470984	0.079472456	0.401378994
MaMADS89	0.141080188	0	0	0	0.28022105	0	0	0	0	0
MaMADS90	0.034293269	0.034973776	0	0.034898824	0	0	0.193020614	0	0	0
MaMADS99	0	0.220362384	0	0	0	1.266120971	0.362258715	0.039609788	0.269343805	0.079766523

Table S4. The characteristics of linked proteins with MaMADS24

from node	to node direction	linked number	Description
MaMADS24	G2-like	2	Ma07_t00650.1;Ma03_t11700.1
MaMADS24	MYB	4	Ma07_t23180.1;Ma09_t28970.1;Ma07_t19720.1;Ma04_t30160.1
MaMADS24	MYB	4	Ma11_t16150.1;Ma10_t10820.1;Ma07_t26530.1;Ma05_t25630.1
MaMADS24	SWI.SNF-BAF60b	1	Ma02_t15240.1
MaMADS24	MaMADS31	1	Ma04_t30020.1
MaMADS24	MaMADS46	1	Ma07_t25120.1
MaMADS24	MaMADS41	1	Ma06_t16260.1
MaMADS24	MaMADS33	1	Ma05_t01150.1
MaMADS24	MaMADS28	1	Ma04_t14430.1
MaMADS24	MaMADS48	1	Ma08_t04270.1
MaMADS24	C2C2-YABBY	1	Ma05_t25970.1
MaMADS24	GRF	4	Ma06_t26200.1;Ma02_t06600.1;Ma06_t03010.1;Ma03_t23910.1
MaMADS24	GRF	4	Ma11_t19960.1;Ma07_t02290.3;Ma01_t20750.1;Ma03_t16030.2
MaMADS24	EIL	1	Ma11_t23440.1
MaMADS24	OFP	5	Ma09_t02570.1;Ma08_t07670.1;Ma06_t27010.1;Ma02_t21780.1;Ma00_t03590.1
MaMADS24	C2H2	6	Ma08_t30790.1;Ma05_t09700.1;Ma11_t10380.1;Ma01_t21780.1;Ma11_t02060.1;Ma03_t26610.1
MaMADS24	BBR-BPC	1	Ma05_t26890.1
MaMADS24	DBE	1	Ma09_t13520.2
MaMADS24	ARF	3	Ma04_t32510.1;Ma11_t01530.1;Ma05_t04740.1
MaMADS24	E2F-DP	1	Ma03_t16730.1
MaMADS24	AP2	2	Ma03_t13350.1;Ma08_t27240.1
MaMADS24	SBP	6	Ma09_t23570.1;Ma07_t22070.1;Ma01_t22850.2;Ma00_t01900.1;Ma11_t18010.1;Ma02_t08090.1
MaMADS24	SWEET	4	Ma05_t01170.1;Ma08_t00630.1;Ma07_t14600.1;Ma06_t10720.1
MaMADS24	NAC	1	Ma06_t32230.1
MaMADS24	bZIP	4	Ma06_t19670.1;Ma02_t02530.1;Ma02_t22400.1;Ma04_t36040.1
MaMADS24	CPP	2	Ma08_t20510.1;Ma09_t26110.1
MaMADS24	MYB-related	1	Ma04_t14900.1
MaMADS24	PLATZ	3	Ma07_t03690.1;Ma09_t31340.1;Ma06_t21430.1

MaMADS24	LIM	1	Ma04_t22790.1
MaMADS24	SNF2	2	Ma04_t31630.1;Ma08_t09770.1
MaMADS24	WRKY	3	Ma07_t27340.1;Ma07_t27330.1;Ma05_t03920.2
MaMADS24	Rcd1-like	1	Ma03_t12440.1
MaMADS24	PHD	3	Ma01_t11640.1;Ma06_t27230.1;Ma06_t27020.1
MaMADS24	C3H	3	Ma02_t18370.1;Ma06_t14230.1;Ma08_t30980.3
MaMADS24	HB	7	Ma04_t20110.1;Ma06_t12120.1;Ma04_t02700.1;Ma03_t07650.1;Ma04_t19480.1;Ma10_t23350.2;Ma05_t01460.2
MaMADS24	zf-HD	4	Ma04_t07880.1;Ma10_t30220.1;Ma04_t14890.1;Ma07_t20450.1
MaMADS24	Tify	6	Ma02_t10180.2;Ma09_t13660.1;Ma03_t16370.1;Ma00_t01940.1;Ma08_t27530.1;Ma08_t04410.2
MaMADS24	SBE	1	Ma06_t15040.2
MaMADS24	C2C2-GATA	3	Ma06_t28060.1;Ma05_t23340.1;Ma04_t10430.1
MaMADS24	LUG	1	Ma07_t08470.3
MaMADS24	CSD	1	Ma04_t22250.1
MaMADS24	ARID	1	Ma03_t11420.1
MaMADS24	HSF	1	Ma06_t00740.2
MaMADS24	ERF	4	Ma01_t23350.1;Ma07_t17760.2;Ma04_t24150.1;Ma06_t06830.1
MaMADS24	B3	4	Ma06_t13580.1;Ma02_t11230.1;Ma01_t22310.1;Ma09_t23900.2
MaMADS24	B3	4	Ma06_t37460.1;Ma08_t13630.1;Ma05_t13970.1;Ma04_t10680.1
MaMADS24	SET	3	Ma07_t17470.3;Ma11_t22910.2;Ma01_t13610.3
MaMADS24	bHLH	7	Ma09_t14430.1;Ma03_t19700.1;Ma11_t16060.1;Ma11_t09210.1;Ma09_t10920.2;Ma08_t19780.1;Ma10_t06760.1
MaMADS24	bHLH	6	Ma10_t23960.1;Ma06_t03850.1;Ma09_t09380.1;Ma11_t00280.1;Ma10_t12250.1;Ma08_t18460.1
MaMADS24	Orphans	1	Ma02_t17840.1
MaMADS24	VOZ	1	Ma01_t21450.1

Table S5. The characteristics of linked proteins with MaMADS49

from node	to node direction	linked number	Description
MaMADS49	NAC	6	Ma06_t33280.1;Ma07_t11300.1;Ma06_t33980.1;Ma02_t10970.1;Ma09_t04890.1;Ma11_t16350.1
MaMADS49	ERF	2	Ma10_t31080.1;Ma06_t09740.1
MaMADS49	Trihelix	4	Ma11_t03370.1;Ma03_t23920.1;Ma04_t31310.1;Ma03_t13610.1
MaMADS49	Orphans	2	Ma11_t13610.1;Ma07_t18180.1
MaMADS49	MaMADS53	1	Ma08_t28680.1
MaMADS49	MaMADS58	1	Ma09_t14960.1
MaMADS49	OFP	3	Ma09_t05360.1;Ma04_t24770.1;Ma08_t12200.1
MaMADS49	bHLH	6	Ma01_t10120.1;Ma05_t17970.1;Ma04_t05320.1;Ma03_t10360.1;Ma02_t08030.1;Ma04_t30910.1
MaMADS49	Tify	2	Ma09_t15040.1;Ma03_t09980.1
MaMADS49	ACO	1	Ma06_t02600.1
MaMADS49	C2C2-GATA	1	Ma11_t10510.1
MaMADS49	PGM	1	Ma02_t08630.1
MaMADS49	FAR1	1	Ma02_t10200.1
MaMADS49	WRKY	4	Ma10_t26000.1;Ma04_t17390.1;Ma07_t01510.1;Ma04_t24790.1
MaMADS49	PHD	2	Ma06_t12770.1;Ma04_t05650.2
MaMADS49	LIM	1	Ma06_t29950.1
MaMADS49	G2-like	2	Ma10_t12790.1;Ma06_t11380.1
MaMADS49	BMY	3	Ma06_t07470.1;Ma05_t07800.1;Ma04_t18390.1
MaMADS49	MYB	4	Ma07_t23230.1;Ma04_t16770.1;Ma04_t34660.1;Ma06_t00910.2
MaMADS49	ACS	1	Ma04_t35640.1
MaMADS49	C2C2-Dof	1	Ma08_t02400.1
MaMADS49	PLATZ	1	Ma03_t14350.1
MaMADS49	IWS1	1	Ma01_t06780.1
MaMADS49	LUG	1	Ma05_t09900.1
MaMADS49	bZIP	7	Ma03_t23820.1;Ma06_t10540.1;Ma11_t23520.1;Ma08_t23040.1;Ma06_t01870.1;Ma10_t15920.1;Ma04_t35070.1
MaMADS49	NF-YC	1	Ma11_t00430.1
MaMADS49	SSS	1	Ma03_t03110.2
MaMADS49	AMY	1	Ma08_t04100.1

MaMADS49	zf-HD	1	Ma08_t09070.1
MaMADS49	LOB	5	Ma04_t13810.1;Ma03_t14170.1;Ma01_t14320.1;Ma07_t23670.1;Ma06_t17210.1
MaMADS49	Rcd1-like	1	Ma01_t19130.2
MaMADS49	AP2	1	Ma07_t18010.1
MaMADS49	G6PT	1	Ma07_t24130.1
MaMADS49	C2H2	4	Ma07_t21790.1;Ma11_t20280.1;Ma09_t03200.1;Ma06_t26490.1
MaMADS49	C2H2	4	Ma10_t15250.1;Ma05_t25510.1;Ma10_t06120.1;Ma04_t20480.1
MaMADS49	HB	3	Ma04_t01270.1;Ma04_t34900.1;Ma03_t27410.1
MaMADS49	TCP	1	Ma06_t01380.1
MaMADS49	C2C2-YABBY	1	Ma08_t27340.1
MaMADS49	GNAT	1	Ma03_t06020.1
MaMADS49	GRAS	3	Ma05_t16130.1;Ma11_t02690.1;Ma05_t12570.1
MaMADS49	B3	1	Ma11_t09870.1

Table S6. The primer sequences used for qRT-PCR

Gene	Forward primers(5'-3')	Reverse primers(5'-3')
MaMADS12	CAGAGCTGGAACACACGCA	CCAGAGCTGCAGGTTATGGC
MaMADS24	TATGCGATGCACAGGTTGGA	GCTTGCCTGGAGCTTATCGT
MaMADS55	AAGCCTACGAGCTTTCGGTG	AGCTCCTTGATGCTGAGTGG
MaMADS73	CTCCTTGGTGAGGACTTGGG	GCTGGATGGGCACTGTTTTC
MaMADS49	CGAGAAGGCCATGAAGAAGG	CTTGGGAACGAAGGACTGTT
MaMADS53	GAATGGCCTGCTGAAGAAGG	TGAAACCATGGCTTCTGATGC
MaRPS2	TAGGGATTCCGACGATTTGTT[illegible]	TAGCGTCATCATTGGCTGGGA
MaUBQ2	GGCACCACAAACAACACAGG	AGACGAGCAAGGCTTCCATT
19 genes interacted with MaMADS24		
MaMADS41-Ma06_t16260.1	CGGATCTGTCCCGCAACTTA	TTTGCAGCTCCATCTCCCTT
C2C2-YABBY-Ma05_t25970.1	TCACCCACAAGGAGGCATTT	CAGGTCTCTTCTCCGGAGCA
GRF-Ma03_t16030.2	TGAATGAAGTGTGCCATCGAC	ACTGCCATTGCTGCTCGAC
OFP-Ma09_t02570.1	GACTCGTTCTTCACCCGCTC	CCATTGACTTGGAGGGTGAAA
C2H2-Ma03_t26610.1	ACGGTGACCTGGGAGCTTT	CCACATTGGCAGGTCTTGG
DBE-Ma09_t13520.2	TCCGAAACCAGCAGATTTGTC	ACAGGTTGGCGTCCAGGAA
ARF-Ma11_t01530.1	TACCAAGCCATGTCCGCTG	TGGTGCGGAGCAGAGTTTG
SWEET-Ma07_t14600.1	TCACAATTCCCAATGGTCTGG	CACATGTCGCGATTCTCCG
NAC-Ma06_t32230.1	CAGCAAGAACGAAGCCTTTCC	GCCGCTCATCTGCTTCTTCAC
WRKY-Ma07_t27330.1	GAGGCCACTCAGAGCAGGTTT	CCTTTGACATGCTTGTGCCC
HB-Ma04_t20110.1	CGGAAGAGAACCGAAGGCTT	ATCGAGGAAGGACGGACGA
zf-HD-Ma04_t07880.1	ATGCCGAGGAAGCGGTTT	CGGTAGCTGCGATTGCTGT
SBE-Ma06_t15040.2	TCAGTCAATATCAGGAGGGCG	TCCCATTGACGGTTTGCC
LUG-Ma07_t08470.3	TGGGATCCTTCTGGTGAGCTT	TGCGATAAGCCCTTCATGTG
CSD-Ma04_t22250.1	CGTCCACCAGTCCTCCATCA	CCGCCTCGATCGTTAAACC
HSF-Ma06_t00740.2	TGACATCGTCTGCTTCATCCA	CTTTGCCGTGGTTGTTTCG
ERF-Ma06_t06830.1	TCCGCCTCGACACAGAGAA	TCTATCATCTGCAACGCCAGC
B3-Ma04_t10680.1	TCAAAGCGTCCTCCTCCGT	CCCTTTCCTTCAGTCGAGAGC
bHLH-Ma11_t09210.1	TGCTTAGATCCGTCGTCCCA	CGGGCAGAGTTCATCCTTCAT
19 genes interacted with MaMADS49		
NAC-Ma06_t33980.1	CAGCAACTGCAGCAATGACA	TGCTGATTGAAGAAGCGCTG
ERF-Ma06_t09740.1	CTTGCAGAATGTAGCTTCGGG	GGAGGTCGACTGCCGTCTTT
Trihelix-Ma04_t31310.1	GCCATGGATGATGACTACGCT	TGGCCAAGAAAGAACCCG
MaMADS53-Ma08_t28680.1	TCAACAGGCAGGTGACGTTC	CCATGGCTTCTGATGCATTG
OFP-Ma09_t05360.1	GAGGTCGGATTCCCTGTGGT	CCCAGGATAGCTTCCCATGTG
Tify-Ma03_t09980.1	ACTTCCATCTTGGCCTCCG	TCCCGGAGCTGTGTTCTTG
ACO-Ma06_t02600.1	ATCAGGTCGAGGTCGTGAGC	CACGAGCGTCTGCTTCATGT
PGM-Ma02_t08630.1	TCTAAGCATCAAGGCATCCGA	CAGTAGGAGCAGATCGGCCA
WRKY-Ma04_t24790.1	CGATAACCGTGACCAGCTGC	AGAACTGGTCCTCGAAGCCG
BMY-Ma04_t18390.1	AGGCGTACTCGCAGGTGTTG	CAGCGGATGCTTCAGCCAT
MYB-Ma07_t23230.1	GCCATGCCCTGTTTCTTGA	ACTCCAACACCCATGAAATCG
ACS-Ma04_t35640.1	TCAAGCGAGTCGGGATTCAT	TCGATCCTCCTCAGCGCTA
bZIP-Ma06_t10540.1	CCCTCAGCATCACCACACAG	GTGGAACATGTCAGAGGAGG[illegible]
SSS-Ma03_t03110.2	TCAACTGCACTGGTGGTCTCA	CGTCCAATCAAAGATCCGCT
AMY-Ma08_t04100.1	TCTTAACGCATCCTGGCACA	GGCTCATAATGTCCTGGCCC

LOB-Ma04_t13810.1	CGTCAACCCGATCTACGGCT	GGGCTTGTTGACCTTGTGGA
G6PT-Ma07_t24130.1	TGTTCAATTCCGCTCTTGCC	TGTTGCGTTTCTTTGCTTCG
C2H2-Ma10_t06120.1	GGTCCACGAGTGCTCCATCT	CCACAACCCACCATTGGAAT
GRAS-Ma05_t16130.1	GACGGGCAGTCAATTCGGT	CCCTTCCATCCGAATCCAA

Table S7. The full names and abbreviations of MADS-box subfamily

Full name	Abbreviation
AGAMOUS	AG
AGAMOUS-LIKE	AGL
APETALA1/ FRUITFULL	AP1/FUL
APETALA2	AP2
BSister	BS
DEFICIENS/GLOBOSA	DEF/GLO
FLOWERING LOCUS C	FLC
SEPALLATA/AGAMOUS-LIKE 2	SEP/AGL2
SOLANUM TUBEROSUM MADS-BOX 11/SHORT VEGETATIVE PHASE	STMADS11/SVP
Tomato MADS-box gene 3-like/SUPRESSOR OF OVEREXPRESSION OF CONSTANT[illegible]	TM3/SOC1

Table S8. The full names of interacted proteins with MaMADS24 and 49

Full name	Abbreviation
1-aminocyclopropane-1-carboxylic acidoxidase	ACO
1-aminocyclopropane-1-carboxylic synthase	ACS
α-amylase	AMY
auxin responsive factor	ARF
A-T rich interaction domain	ARID
the third basic domain	B3
basic pentacysteine	BBR-BPC
basic helix-loop-helix	bHLH
β-amylase	BMY
the basic region/leucine zipper motif	bZIP
C2 C2 zinc finger-YABBY	C2C2-YABBY
C(2)-C(2) zinc finger DNA-binding with one finge	C2C2-Dof
Cys2His2 zinc finger protein	C2H2
Cys3His zinc finger domain	C3H
cystein-rich polycomb-like protein	CPP
cold shock domain	CSD
de-branching enzyme	DBE
E2F4-DP2- DNA complex	E2F-DP
ethylene insensitive 3-like	EIL
ethylene-responsive factor	ERF
far-red-impaired response	FAR1
Golden2-like	G2-like
glucose-6-phosphate transferase	G6PT
Bsister	GGM13
GCN5-related N-acetyltransferase	GNAT
GAI, RGA and SCR	GRAS
growth regulating factor	GRF
heat shock factor	HSF
interact with Spt6	IWS1
lin-11, isl-1, and mec-3	LIM
lateral organ boundaries	LOB
LEUNIG	LUG
myeloblastosises	MYB
NAM, ATAF and CUC	NAC
nuclear factor Y subunit C	NF-YC
ovate family protein	OFP
phosphorglucomutase	PGM
plant homeodomain	PHD
plant AT-rich sequence and zinc-binding protein 1	PLATZ
the radical induced cell death protein 1-like	Rcd1-like
starch branching enzyme	SBE
squamosa promoter-binding protein	SBP
Su (var), E (z), and Trithorax	SET
sucrose nonfermenting 2	SNF2
sugars will eventually be exported transporters	SWEET
soluable starch synthase	SSS
TB1, CYC and PCFs	TCP
[TIF[F/Y]XG	Tify

helix-loop-helix-loop-helix	Trihelix
vascular plant one-zinc finger	VOZ
WRKYGQK	WRKY
zinc finger-homeodomain	zf-HD
